# Experimental parameters defining ultra-low biomass bioaerosol analysis

**DOI:** 10.1038/s41522-021-00209-4

**Published:** 2021-04-16

**Authors:** Irvan Luhung, Akira Uchida, Serene B. Y. Lim, Nicolas E. Gaultier, Carmon Kee, Kenny J. X. Lau, Elena S. Gusareva, Cassie E. Heinle, Anthony Wong, Balakrishnan N. V. Premkrishnan, Rikky W. Purbojati, Enzo Acerbi, Hie Lim Kim, Ana C. M. Junqueira, Sharon Longford, Sachin R. Lohar, Zhei Hwee Yap, Deepa Panicker, Yanqing Koh, Kavita K. Kushwaha, Poh Nee Ang, Alexander Putra, Daniela I. Drautz-Moses, Stephan C. Schuster

**Affiliations:** 1 Singapore Centre for Environmental Life Sciences Engineering (SCELSE), Nanyang Technological University, Singapore, Singapore; 2 Present Address: Departamento de Genética, Instituto de Biologia, Universidade Federal do Rio de Janeiro, Rio de Janeiro, 21941-590 Brazil

**Keywords:** Next-generation sequencing, Environmental microbiology

## Abstract

Investigation of the microbial ecology of terrestrial, aquatic and atmospheric ecosystems requires specific sampling and analytical technologies, owing to vastly different biomass densities typically encountered. In particular, the ultra-low biomass nature of air presents an inherent analytical challenge that is confounded by temporal fluctuations in community structure. Our ultra-low biomass pipeline advances the field of bioaerosol research by significantly reducing sampling times from days/weeks/months to minutes/hours, while maintaining the ability to perform species-level identification through direct metagenomic sequencing. The study further addresses all experimental factors contributing to analysis outcome, such as amassment, storage and extraction, as well as factors that impact on nucleic acid analysis. Quantity and quality of nucleic acid extracts from each optimisation step are evaluated using fluorometry, qPCR and sequencing. Both metagenomics and marker gene amplification-based (16S and ITS) sequencing are assessed with regard to their taxonomic resolution and inter-comparability. The pipeline is robust across a wide range of climatic settings, ranging from arctic to desert to tropical environments. Ultimately, the pipeline can be adapted to environmental settings, such as dust and surfaces, which also require ultra-low biomass analytics.

## Introduction

Great naturalists of centuries-past have catalogued planetary ecosystems at the macroscopic level, primarily for terrestrial and aquatic environments, where organisms were most accessible^1,2^. Microscopic life was subsequently given the same attention, again initially focusing on terrestrial and aquatic systems^3,4^. Microbial inhabitants of the third ecosystem of planetary scale, the atmosphere, proved much more difficult to assess due to technological challenges in regard to accessibility. These challenges are largely associated with the low-density gaseous state and resulting ultra-low biomass of air^5–7^. As a consequence, atmospheric research first described the physicochemical nature of the atmosphere, thereby generating a comprehensive understanding of inanimate components of the troposphere and stratosphere^8^. The origin of these components of air is typically categorised as either inorganic gases or volatile organic compounds (VOCs), the latter of which serve as proxies for the biological activity of organisms^9,10^. The following progression in the field involved the identification of airborne organisms via cultivation and microscopy^11,12^. This provided a foundation for understanding the composition of airborne microbial organisms via nucleic acid taxonomic identification. A large increase of the taxonomic resolution was subsequently achieved by the use of ITS and 16S rRNA gene markers. The ultra-low biomass nature of air posed major technical obstacles to using these molecular techniques, with inherent requirements such as long sampling duration and high amounts of gene marker amplification^13–18^.

The nascent field of bioaerosol studies was further progressed by employing metagenomics, which enabled direct nucleic acid analysis without the biases associated with gene amplification. However, to overcome issues associated with limited biomass, long sampling duration times (days to weeks) were unavoidable, which in turn impeded the temporal resolution and the number of required samples analysed^19–21^.

Advances in temporal and taxonomic resolution only became possible with the onset of new technologies involving high volumetric flow rate air samplers coupled with metagenomic data generated by next-generation sequencing platforms that had low biomass requirements^22^. This approach, which analyses the accessible spectrum of airborne community DNA, therefore enables assessment of the functional complement of airborne microorganisms.

Here, we detail optimisation of multiple stages of an ultra-low biomass analysis pipeline for air samples, which can also be tailored to studies of similarly ultra-low biomass environments such as dust and surfaces. The versatility and robustness of the presented pipeline enable analysis of a wide range of environmental settings, both indoor and outdoor, encompassing a wide scope of climatic settings including tropical, temperate, desert and arctic regions.

## Results

### Environmental samples: soil, water, air

**Fig. 1:**
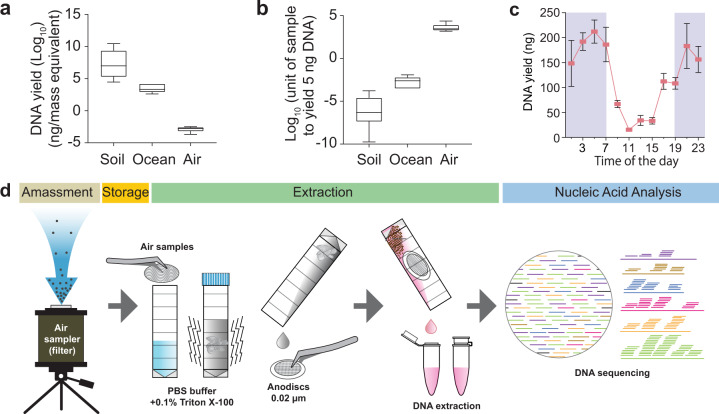
Challenges in air microbiome analysis. **a** Total DNA yield (ng/mass equivalent) for soil, ocean water and air sample collected from the same proximity and processed with the same method. **b** estimated sample volume required to yield 5 ng of DNA. For box plots, the centre line, bound of box and whiskers represent median, 25th–75th percentile and min-to-max values, respectively. **c** Fluctuation of airborne biomass (ng) at different times of the day. The red dots and error bars are mean and standard deviation among the replicates. **d** Developed sampling and analysis pipeline for metagenomic analysis of ultra-low biomass environmental samples.

Ecosystems and habitats are highly variable and complex, and hence a universal approach is not always applicable. Using DNA concentration as a proxy, terrestrial, aquatic and atmospheric ecosystems can harbour up to a six-log difference in microbial biomass (Fig. 1a). This results in vastly different sampling requirements and volumes for molecular analysis (Fig. 1b). In addition, biomass concentrations might follow cyclic processes resulting in density fluctuations, as shown in marine environments^23^ as well as atmospheric environments where higher bioaerosol concentrations are typically observed at night^22^ (Fig. 1c) or during haze events^24^.

**Fig. 2:**
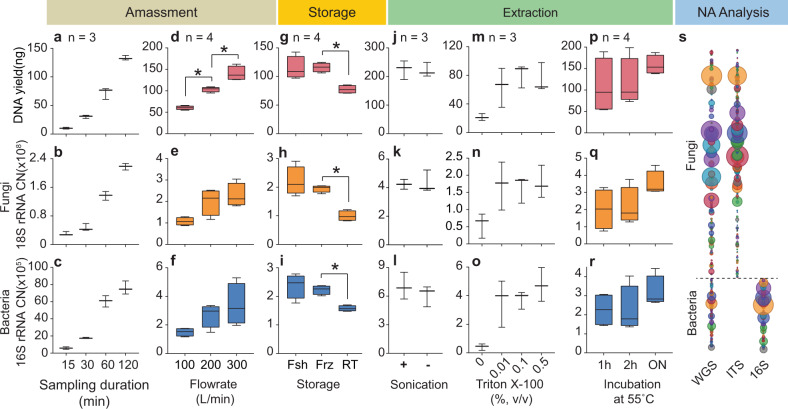
Summary of quantitative analysis with DNA yield, 18S copy number (CN) and 16S copy number (CN). **a**–**c** Assessment of air sampling duration from 15 min to 3 h. **d**–**f** Assessment of air sampling flow rate from 100 L/min to 300 L/min. **g**–**i** The integrity of sampled biomass when processed fresh (Fsh), stored in freezer for 5 d (Frz) or stored at room temperature for 5 d (RT). **j**–**l** Impact of sonication on DNA yield. (**m**–**o**) Impact of detergent addition at different concentrations (0.01–0.5% v/v) during filter sample wash. **p**–**r** Impact of extended pre-incubation (1 h to overnight) at 55 °C during DNA extraction. The centre line, bound of box and whiskers represent median, 25th–75th percentile and min-to-max values, respectively. **s** Whole-genome shotgun (WGS) and amplicon (ITS/16S) sequencing approaches. * denotes statistical significance (*p* < 0.05) tested with Mann–Whitney tests.

To address the challenges in analysing a wide range of biomass concentrations at different spatial and temporal settings, we developed a robust ultra-low biomass pipeline, comprising the four-stages of amassment, storage, extraction and nucleic acid analysis. Parameters that impact upon the pipeline’s efficacy were investigated, with the aim of enabling customisation (Fig. 1d). The summarised results are displayed in Fig. 2. The subsequent sections detail each investigated parameter individually.

### Amassment

The ultimate success of sequencing and PCR-based analyses rests on sufficient quantities of nucleic acids being amassed, which for air sampling is a trade-off between sampling flow rate and sampling duration. While this study uses a filter-based sampler, other types of air samplers, such as liquid impingers serve a similar function and produce comparable results (Supplementary Fig. 8)^25^. For our purpose, ideal air samplers should be portable, battery-powered and have an acceptable noise emission (~50 dB).

**Fig. 3:**
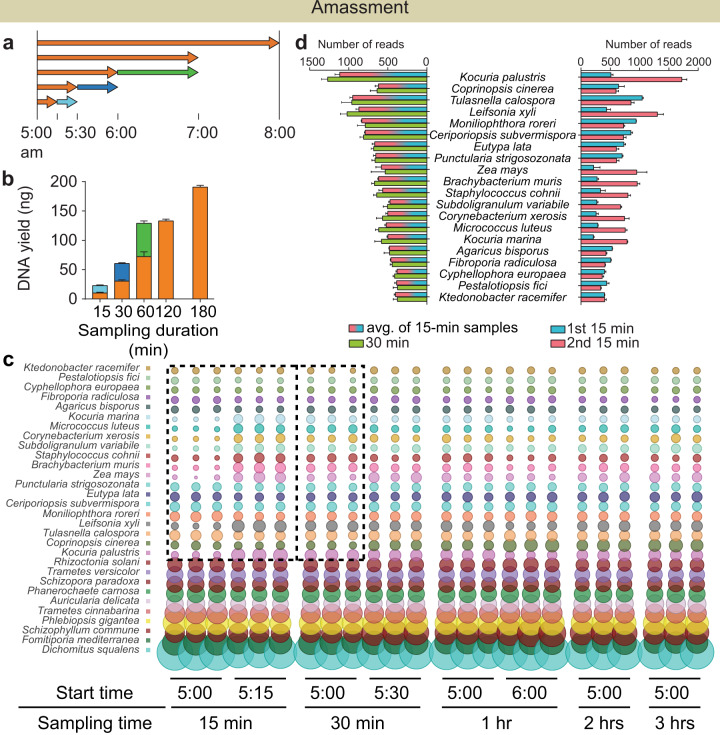
Sampling duration assessment. **a** Illustration of different time-based sampling regimes. **b** Comparison of DNA yield (ng) between the corresponding sampling regimes, e.g. first 15-min yield (orange) + second 15-min yield (light blue) compared to first 30-min yield (orange). The bars represent mean values and the error bars were standard deviation among the replicates. **c** Taxonomic compositions of the top 30 species, the highlighted portion focuses on species which shifted in abundance between the first and second 15-min samples. **d** Comparison of relative abundances of the selected species, the first and second 15-min samples were averaged and compared to the taxonomic composition of first 30-min sample. The bars represent mean values and the error bars were standard deviation among the replicates.

The air sampling flow rate and duration were optimised to improve the temporal resolution of each sample from days, weeks or months to hours or even minutes, while still maintaining maximal taxonomic resolution. This was achieved by evaluating how these two factors directly impact the DNA quantity and metagenomic profile of the sample. Using 300 L/min flow rate, the minimal required sampling duration was investigated using different time-based sampling regimes (Fig. 3a). Sampling duration was segmented into sequentially doubling time intervals. For example, the first and second 15-min intervals (5:00–5:15 am and 5:15 to 5:30 am) were individually analysed and compared to a 30-min sample (5:00–5:30 am) taken in parallel. This process was undertaken for 15, 30 and 60-min intervals with a final sampling duration up to 180 min. Quantitative analysis showed consistently increasing DNA yields as a function of sampling duration (Fig. 2a–c). No notable loss of DNA yield was observed within the tested range of duration (15 min–3 h). Within this range, combining two successive time segments resulted in similar DNA quantities as a single time segment of the combined duration, as quantified using Qubit and qPCR (Fig. 3b, Supplementary Fig. 1). Within the three investigated intervals (three duration groups each for Qubit, bacterial and fungal qPCR), the differences averaged 25%, with a median of 18%. Importantly, the microbial taxonomic profiles from comparable time intervals were not affected. This is demonstrated by the shift in relative abundances of taxa, such as *Kocuria palustris*, and *Leifsonia xyli*, between the two subsequent 15-min samples (Fig. 3c). Averaging these species compositions from the two subsequent 15-min filter samples resulted in abundances that mirror that of the 30 min time interval sample collected in parallel (Fig. 3d). This was consistent across all sampling duration regimes with three replicates each (BrayCurtis and Jaccard *p* > 0.05).

The second experiment examined the impact of the air flow rate and the total volume of air sampled. With the sampling duration set at 2 h, airflow was varied between 100, 200 and 300 L/min, resulting in total air volumes of 12, 24 and 36 m^3^, respectively. The DNA yield and copy number of marker genes (16S and 18S rRNAs) increased as a function of air volume sampled (Fig. 2d–f). However, DNA concentration normalised per air volume diminished by up to 20% when the flow rate was increased from 100 to 300 L/min (Supplementary Fig. 2a). The diminishing return of amassment is likely due to decreasing particle retention efficiency at higher flow rates for extended periods of time^26^. For the purpose of this study, optimal sampling efficiency is forfeited in favour of higher flow rates (300 L/min) because the total amount of biomass collected per unit of time still out-performs the decrease in amassment efficiency. This enables measurements with higher time resolution within a day for environmental time-series studies. The biological significance of this was demonstrated by the discovery of diel dynamics of outdoor airborne microbial communities^22^.

Further analysis demonstrated that flow rate does not impact the qualitative and quantitative assessment of metagenomic data (Supplementary Fig. 2b). The community structure (BrayCurtis, *p* > 0.05) and richness (Jaccard, *p* > 0.05) were not significantly different for samples collected with different flow rates.

### Storage

Analysis of the storage component in this pipeline evaluated the integrity (biomass quality and composition) of air filter samples stored under different conditions. The three conditions investigated were (i) instant processing (Fsh), (ii) 5-day storage at −20 °C (Frz), and (iii) 5-day storage at room temperature (RT, average 23 °C, RH 65%).

**Fig. 4:**
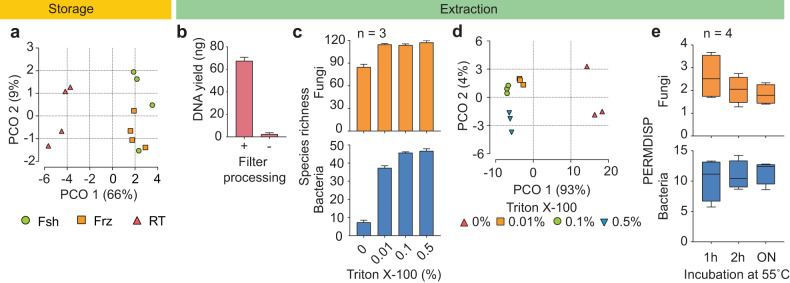
Storage and biomass extraction. **a** Principal coordinate analysis (Bray-Curtis) on genus level for samples processed fresh (Fsh), stored in freezer (Frz) and room temperature (RT). **b** Comparison of DNA yield (ng) with (+) and without (−) the filter wash step. **c** Total identified species for fungi (orange) and bacteria (blue) for samples processed with different concentration of detergent (0–0.5% v/v) during the wash step. The bars represent mean values and the error bars were standard deviation among the replicates. **d** Principal coordinate analysis (Bray-Curtis) on genus level for samples processed with different concentration of detergent (0–0.5% v/v) during the wash step. **e** PERMDISP analysis for samples processed with extended incubation at 55 °C prior to cell lysis. The centre line, bound of box and whiskers represent median, 25th–75th percentile and min-to-max values, respectively.

No significant differences were observed between fresh and freezer samples in terms of both absolute (Qubit, qPCR) and relative (metagenomic) abundances. This suggests temporary freezer storage is a viable alternative to immediate filter processing. However, RT samples were significantly different from the fresh and freezer regimes in regard to DNA quantities (20–30% loss) (Fig. 2g–i). Also, a minor decrease of relative abundance of certain taxa was observed (BrayCurtis, *p* < 0.05) (Fig. 4a); however, there was no loss in the number of species detected (Jaccard, *p* > 0.05) (Supplementary Fig. 3). This outcome implies that microbial growth on the filter substrate is impeded within the course of several days, thus enabling sample collection for field surveys without the need for refrigeration^27^.

### Filter processing and DNA extraction

As library construction for DNA sequencing requires the removal of particle/biomass from the air filter substrate (referred to as filter processing), the protocol was optimised for efficient biomass retrieval. Importantly, the ultra-low biomass nature of the sample renders filter processing the most limiting, and hence, the most critical step across the entire pipeline for maximising yield.

In general, filter samples can be processed in one of two ways, either direct DNA extraction on the filter, or by first removing the biomass from the filter prior to DNA extraction. Direct DNA extraction was deemed inefficient as the filter absorbs most of the lysis buffer, which consequently inhibits cell lysis. In contrast, first removing the biomass by washing the filter in a buffer (PBS) and then concentrating on a thinner membrane with smaller mesh-size (0.2 µm PES or Anodisc membrane)^28^, resulted in significantly higher DNA recovery (Fig. 4b).

To further improve biomass recovery, additional steps such as water-bath sonication (RT, 1 min)^29,30^ and the use of detergent (Triton-X 100) during filter wash were tested. For comparison of samples processed with and without sonication, no significant difference in either quantitative or metagenomic analyses was found (Fig. 2j–l and Supplementary Fig. 4). In contrast, adding detergent during the filter wash significantly improved DNA yield (Fig. 2m–o). The hydrophobic nature of the air sampling filter impeded wetting by the wash buffer. Hence, particles were not effectively suspended in the wash buffer when mechanically agitated. The addition of non-ionic detergent, Triton X-100, at varying concentrations (%v/v) (PBS-T) to the initial PBS buffer was effective in overcoming this challenge.

The detergent wash resulted in significant differences in absolute and relative abundance analyses, especially in the instance of bacteria. DNA yield, as well as copy number of bacterial 16S and fungal 18S rRNA genes, increased 2.4, 8.6 and 2.0-fold, respectively (Fig. 2m–o). The metagenomic analysis confirmed this finding (BrayCurtis and Jaccard, *p* < 0.05, Fig. 4c, Supplementary Fig. 5). The number of detected bacterial taxa increased eight-fold compared to a 1.3-fold increase in fungal taxa. Expectedly, PBS-T treated samples also showed greater taxonomic diversity (Fig. 4c).

Varying concentrations of Triton X-100 (0.01, 0.1 and 0.5% (v/v)) in PBS were investigated, with no significant difference between the three concentrations for quantitative analyses (Fig. 2m–o). However, metagenomic analysis identified notable differences in microbiome composition (BrayCurtis *p* < 0.05, Supplementary Fig. 5a–b) driven by an increase in bacterial taxa. Increasing Triton X-100 beyond 0.1% concentration, yielded no significant further gains (Fig. 4d). Hence, Triton X-100 at 0.1% was deemed sufficient for wetting the filter and releasing attached bioaerosol particles into the buffer medium. Despite the 0.1% concentration of Triton X-100 being above the critical micelle concentration^31^, Triton X-100 did not trigger unwanted premature lysis of microbial cells, as there were no significant differences in DNA yield between the three concentrations. If premature lysis occurred, extracellular DNA would not have been retained on the subsequent Anodisc membrane, resulting in lower DNA recovery.

Following filter processing, the recovered biomass was filtered through a 0.02 µm pore-sized Anodisc membrane (Whatmann, USA) mounted on a vacuum manifold (Fig. 1d), with the Anodisc directly fitting into the DNA extraction kit bead tube. DNA extraction used the standard protocol of the extraction kit with slight modification to improve lysis^26^. In this regard, the addition of overnight pre-incubation of the samples at 55 °C is recommended as it improves evenness among the samples, especially for the representation of fungal taxa, as shown by the quantitative and PERMDISP analysis (Fig. 2p–r, Fig. 4e).

### Nucleic acid analysis of ultra low biomass samples

The outcome of the above sample processing pipeline results in double-stranded DNA samples (in the range of 0.1–7.1 ng DNA/m^3^ of air sampled). These can subsequently be analysed, not only by amplification-based techniques (16S/ITS), but also via direct DNA sequencing (shotgun), resulting in either gene-based or metagenomic profiles of airborne environmental communities. Both approaches, 16S/ITS amplicon and whole-genome shotgun metagenomic (WGS), produce sequence data that may be compared against publicly available data archives. For the remainder of this manuscript, the advantages and disadvantages of both techniques will be discussed in relation to ultra-low biomass analysis.

Amplicon-based sequencing approaches have been the method of choice in the majority of past bioaerosol studies^13–18^. This was due to the assumption that the low amount of amassable biomass from air was insufficient for shotgun metagenomics^32^. Our study shows that the above-described ultra-low biomass air sampling and processing pipeline is capable of robustly producing metagenomic datasets, as demonstrated i) for a range of DNA input amounts, ii) by the reproducibility among replicates, iii) by the robustness of air samples analysis from various climatic conditions and iv) contamination control.

**Fig. 5:**
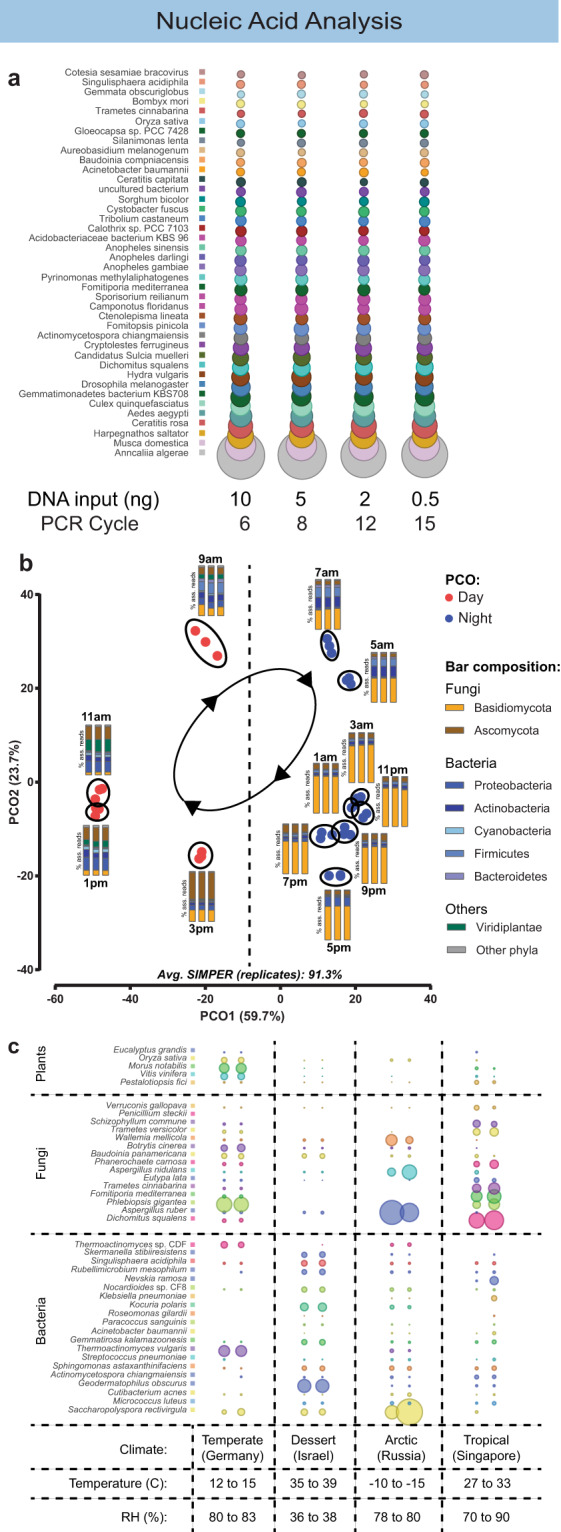
Whole genome shotgun (WGS) sequencing of air samples. **a** Comparison of taxonomic profile at species level for the same air sample that was subjected to WGS sequencing with different DNA input amounts (10–0.5 ng). **b** Reproducibility of samples collected at the same time and location (triplicates) illustrated in principal coordinate analysis (Bray–Curtis) at species level. The bars show the microbial community composition of the triplicates in % of assigned reads. **c** Robustness of air sampling and processing pipeline tested at locations with temperate, dessert, sub-arctic and tropical climates.

Required input amount: from the same DNA sample, a range of DNA input amounts for shotgun metagenomic sequencing and analysis (0.5–10 ng) were tested. Using our pipeline, taxonomic representation for each DNA input condition was visualised at the species level using bubble charts (Fig. 5a). For the tested range of 0.5–10 ng, no significant change of species-level composition was observed. The species-level metagenomic profile for each sample was consistent even when the PCR cycles required during DNA library construction were increased from 6 to 15 (Fig. 5a).

Reproducibility: An experimental time series of outdoor air in a tropical setting^22^ was used to assess sample-to-sample variability. Over 24 h, air samples were collected at 2-h time intervals (12 time points) in triplicate. The metagenomic profiles of samples within the same replicate group were highly consistent, with an average similarity of 91% (87–95%, SIMPER analysis). The taxonomic profiles, however, were distinct between sampling time points (Fig. 5b). The higher variability observed for day-time samples can be attributed to increased atmospheric turbulence due to convection, while a narrower range was observed during night-time hours.

Robustness: The above-tested range of 0.5–10 ng of DNA templates, with their respective PCR cycles (15 to 6 cycles), was suitable for a global air microbiome survey that involved a wide range of environmental conditions. The pipeline presented here robustly produced metagenomic datasets from air samples collected in locations with a diverse range of temperature (−10 to 39 °C) and humidity (36–90%), within the four climatic zones (temperate, dessert, sub-arctic and tropical) (Fig. 5c).

Contamination control: The negative controls consisted of filter blanks (clean, unused filter) mounted on the air samplers for 1 min without airflow, which were then transported and processed in an identical manner to air samples. The DNA yield from negative controls was not detectable (Supplementary Fig. 7a). The number of reads generated by Illumina sequencing were on average 1000-fold less for the negative controls compared to the air samples (Supplementary Fig. 7b), with taxonomic analysis indicating human contamination as the most likely source (Supplementary Fig. 7c). The number of reads from our air samples which could be mapped back to the filter blanks were very low and they were removed by our statistical analysis threshold (<0.05% of assigned reads). It can be deemed that despite the ultra-low biomass nature of our analytical pipeline, contamination is not a concern (Supplementary Discussion 7).

**Fig. 6:**
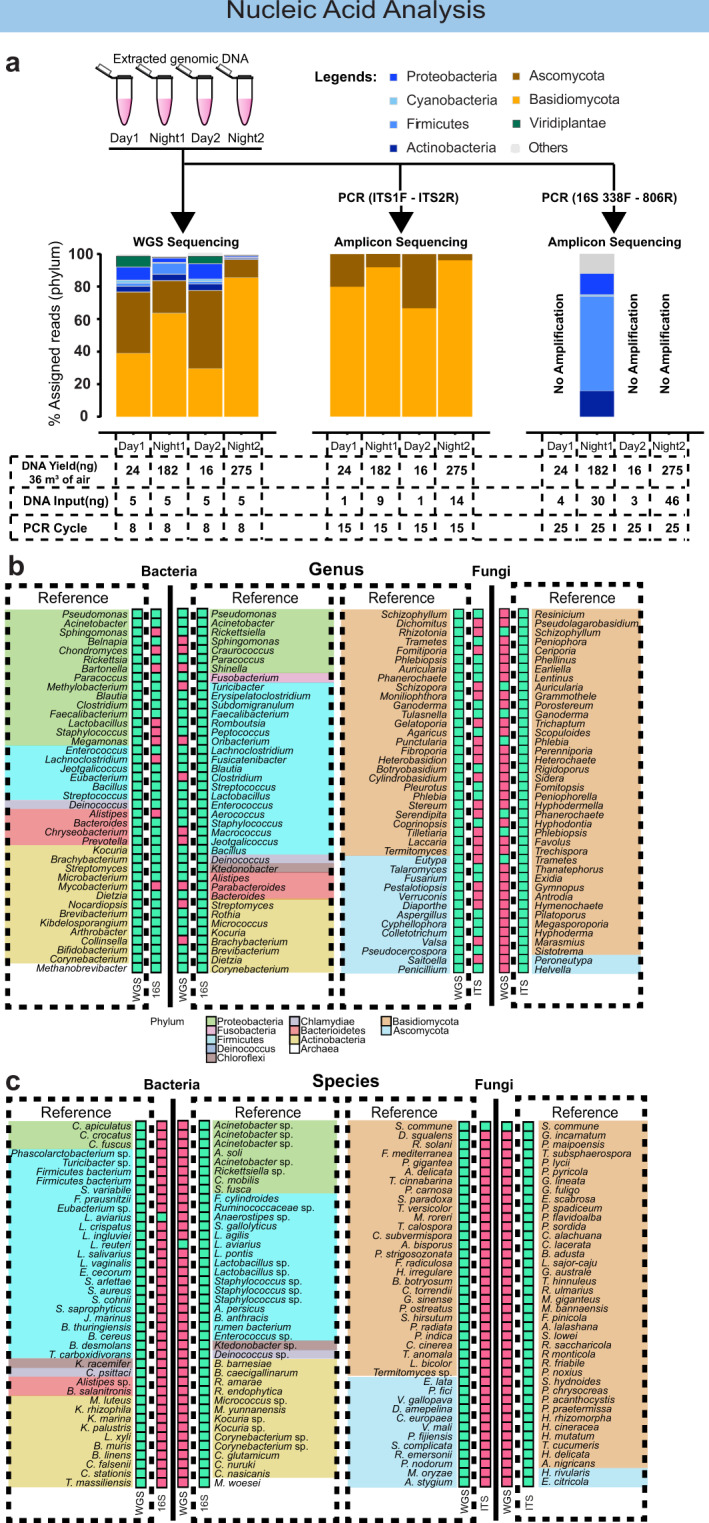
Comparison of taxonomic profiles between WGS and amplicon sequencing pipelines. **a** Taxonomic profile of WGS, ITS amplicon and 16S amplicon pipeline at phylum level of four independently collected air samples (two days and two nights). **b** Presence–absence comparison of the top 40 most abundant genus for bacteria (WGS vs 16S) and fungi (WGS vs ITS). **c** Presence–absence comparison of the top 40 most abundant species for bacteria (WGS vs 16S) and fungi (WGS vs ITS).

In a final step, extracted genomic DNA from the pipeline was analysed by both metagenomic and 16S/ITS amplicon sequencing, resulting in sets of distinct taxonomic profiles based on their respective databases (Fig. 6a). For fungi, results from both sequencing analysis methods concur with the observed trends for the specific abundances of microbial taxa during day/night at higher taxonomic resolution, e.g., Ascomycota being prevalent during day-time and Basidiomycota during night-time. The 16S amplicon analysis, however, was less robust as three out of four samples resulted in no detectable PCR product, even with higher DNA input (4–46 ng) and additional PCR cycles (Fig. 6a). This was caused by low amounts of 16S rDNA gene template in tropical air samples (Supplementary Fig. 9). The only successfully analysed 16S sample resulted in a similar taxonomic profile to that of the WGS pipeline at the phylum level, with Firmicutes dominating over Actinobacteria and Proteobacteria.

The above analysis highlights biases in the success rate of fungal ITS and/or bacterial 16S amplification for air samples from a diverse range of environmental conditions. Numerous studies have reported similar challenges^13,16^. In contrast, regardless of potential inhibitor content and/or taxonomic composition of the air samples, the WGS pipeline consistently captured the biological diversity of airborne microbial communities in various climatic conditions (Figs. 5c, 6a). Moreover, unlike the single gene amplicon approach, the WGS pipeline directly compared DNA read abundances from a diverse set of taxa (bacteria, fungi, plants and others) at a single quantitative scale.

In contrast to phylum level analysis, WGS and amplicon analytical pipelines are substantially less congruent at the genus or species level, due to the respective database sizes. Our metagenomic reads were aligned to the non-redundant (nr) database and assigned to taxa using the MEGAN software^33^, while the amplicon reads were aligned to the 16S SILVA database for bacteria^34^ and ITS UNITE^35^ database for fungi using blastn^36^. The resulting taxonomic classifications from the two analysis approaches show significant agreement at higher taxonomic levels (e.g., up to phylum level). At genus and species levels, taxonomic concordance is diminished, as shown for the top 40 most abundant taxa for both analysis types (Fig. 6b, c). In this regard, the metagenomic and 16S amplicon approach agree in 72–78% of instances on the genus level. However, only one out of 40 taxa (2.5%) was in agreement on the species level. This concordance is even less for fungal taxonomy. On the genus level, 19 out of 40 taxa (47.5%) were in agreement when the WGS was used as a reference. When the ITS was chosen as a reference, seven out of 40 (17.5%) assignments were in agreement. As observed for bacteria, only 1 out of 40 taxonomic assignments was shared on a species level. In general, the amplicon databases possess a much larger representation of fungal and bacterial taxa. The higher overlap for bacteria was likely due to higher representation of bacterial genomes in the nr sequence database due to increasing accessibility for generating genome-wide data for small microbial genomes. In contrast, the accessibility does not extend to genome sizes exceeding 100 MB for some fungal organisms. In this regard, the sequencing, assembly and annotation of fungal genomes are still challenging.

rDNA sequences generated by both sequencing methods concur when analysed for marker gene content. In this regard, the metagenomic datasets analysed in this study contain about 1% rDNA genes (ITS and 16S), which can be aligned to 16S SILVA and ITS UNITE databases. The metagenomics rDNA read analysis and the amplicon sequencing results produced highly overlapping taxonomic profiles for the top 40 most abundant taxa for fungi and the top 10 most abundant taxa for bacteria (Supplementary Fig. 6). With metagenomic sequencing becoming more accessible, it is therefore possible to combine the benefits of 16S- and ITS-based taxonomy to investigate understudied ultra-low biomass environments, while simultaneously enabling taxonomic and functional analyses^37^.

## Discussion

The here-presented air sampling and analysis pipeline enable qualitative and quantitative assessment of microbial diversity in an ultra-low biomass ecosystem. The 5–7 log difference in biomass concentration of air samples, compared to seawater or soil, requires sufficiently large volumes of air to be sampled. Based on our optimisation results, we propose default sampling parameters of 300 L/min for 2 h. This enables DNA accumulation rate which is ~8–170-fold higher than reported in recent studies^26,28,38^ (Supplementary Table 1). This large improvement allows for shorter sampling time (≥15 min), while still enabling WGS metagenomic analysis with species-level taxonomic classification. Such high temporal and taxonomic resolution are crucial for ecological studies of air microbiomes, which rapidly respond to diel dynamics or sudden environmental changes. It should be noted that factors such as time of sampling within a day, sampling duration and climatic settings of the sampling location impact the analysis outcome, and therefore comparability between the above studies. Our proposed method, however, has also been evaluated for its robustness across a wide range of environmental settings (arctic, desert, temperate and tropical climatic zones) (Fig. 5c).

Further, our results indicate that biomass amassed from air samples using filter-based devices during remote fieldwork may be stored at room temperature for extended periods of time with tolerable loss of extractable DNA (20% in 5 d) and without compromising microbial community structure. While these effects could potentially be counter-acted by nucleic acid stabilisation methods^39^, this approach is not recommended during sampling campaigns, as it would require additional handling of the air filter samples in the field. This could result in contamination and complicate transport due to the introduction of liquid materials (e.g., commercial air travel). The advantage of dry storage and transport also does not extend to other types of air samplers, such as liquid impingers.

For nucleic acid extraction, it could be shown that the amassed biomass should not be extracted directly on the filter, but rather first be removed owing to the adherence of the low quantities of nucleic acids to the large surface area of the filter membrane. Therefore, extraction and wash buffer conditions should be optimised to enable the extraction of sub-nanomolar concentrations of DNA/RNA. This optimisation includes the use of detergent and extended incubation times. In particular, the addition of non-ionic detergents, such as Triton X-100, significantly increases the recovered biomass, while extended incubation times improve the evenness of the large sets of samples. This observation is also highly relevant in the context of sampling potentially infectious biological materials, such as airborne retroviruses, which can concurrently be inactivated with Triton X-100^40^.

Both metagenomic and amplicon sequencing methods can be applied to air samples (Fig. 2s). The metagenomic approach is advantageous with regards to enabling simultaneous functional and taxonomic analysis and has the advantage that bacteria and fungi can be analysed within the same quantitative scale. Further, the rapid expansion of the public WGS databases continues to enable species-level taxonomic identification at an increasing rate. In contrast, the content of amplicon sequencing databases (ITS or 16S) are likely to grow at a slower rate, given the increased accessibility of WGS.

While our study demonstrated that the extracted DNA from the ultra-low biomass pipeline was sufficient for WGS and ITS amplicon analyses, 16S amplicons did not perform equally well for tropical air samples (Fig. 6a). This may be due to the fact that the DNA library construction for WGS is less sensitive to inhibitors and the relative ratio of bacterial vs. fungal DNA. Both factors impact on the efficacy of the polymerase chain reaction. Nevertheless, specific gene marker/amplicon analysis can be advantageous for studies that target well characterised, less diverse microbial communities.

Finally, due to database biases, both methods appear to converge on the phylum level, but to a lesser degree at the genus level. On the species level both methods do not produce significant agreement. To harness the advantages from both sequencing technologies, it is beneficial to combine both approaches by also analysing the rDNA sequences from the metagenomic data (Supplementary Fig. 6). The results from this combined analysis enable data interpretation from a single data source (metagenomic data), to inform both WGS and marker genes analysis pipelines.

The here-presented methodology is limited by the size range of the chosen filter medium (0.5− >10 µm, <50% efficiency for particles <0.5 µm). As this study aims to reduce required sampling times, total suspended (biological) particles (TSP) need to be collected and analysed. While this study does not profile particle size range, recent studies have demonstrated that the most relevant airborne bacteria and fungi fall within the size range of the filter medium^13,15^.

In summary, the above-described ultra-low biomass analysis pipeline provides detailed insights into the factors that influence analysis outcomes for low-biomass microbial environments. High volumetric air sampling techniques in combination with applied nucleic acid analysis, results in high temporal and taxonomic resolution of inherent airborne microbial communities. The presented findings are potentially also applicable to other low-biomass environments, such as dust and surfaces.

## Methods

### Air sampling

Air samples for optimisation purposes were collected in Singapore at a roof-top balcony of a university building (N1.346247, E103.679467). As the study focuses on improving the time resolution of the analysis, a high-flow rate, filter-based air sampler (SASS3100, Research International, USA) was used to collect total suspended particles (TSP) with no size cut-off. The filter medium was SASS Bioaerosol electret filter (6 cm diameter, expected 50% efficiency for 0.5 µm particle size, Research International, USA). For sample collection, air samplers were attached upright on a tripod 1.5 m above the concrete floor of the balcony.

In addition to Singapore, samples from different climatic settings were collected in a consistent manner from sites in Germany, Russia and Israel to test the robustness of the proposed pipeline. These international locations showed contrasting settings for temperature (T) and relative humidity (RH).

After sampling, the filters were returned to their original filter pouches and transported to the laboratory for direct processing or storage at −20 °C. Information on exact sampling time, flow rate, duration and the environmental parameter measurements of all sampling activities used in this study can be found in Table 1.

**Table 1:** Details of sampling activities.

Sample set	Sampling date	Sampling time, duration and flow rate	Temperature (°C)	RH (%)	Rain	Sample size	No. of samples
Singapore							
1	29-Nov-17	01:00–03:00 (2 h, 100 L/min, 200 L/min, 300 L/min)	24.8–25.3	98–100	No	4	12
2	15-Dec-17	05:05–08:05 (15 min, 30 min, 1 h, 2 h, 3 h, 300 L/min)	24.7–25.7	99–100	No	3	27
3	29-Nov-17	06:15–08:15 (2 h, 300 L/min)	24.6–25.4	99–100	No	4	12
4	23-Feb-17	17:00–17:00 (2 h, 300 L/min)	24.0–34.0	63–100	Yes	3	36
	24-Feb-17						
5	8-May-16	17:00–17:00 (2 h, 300 L/min)	28.0–33.0	59–89	No	3	36
	9-May-16						
6	29-Nov-17	03:50–05:50 (2 h, 300 L/min)	24.5–24.8	99–100	Yes	3	12
7	28-Nov-17	20:40–22:40 (2 h, 300 L/min)	24.9–25.3	98–99	No	3	12
8	24-Nov-17	05:00–07:00 (2 h, 300 L/min)	23.9–24.5	99–100	No	4	12
9	22-Nov-17	23:00–01:00 (2 h, 300 L/min)	26.0–26.5	97–99	No	3	3
10	23-Nov-17	11:00–13:00 (2 h, 300 L/min)	29.0–30.0	77–80	No	2	2
11	27-Nov-17	23:00–01:00 (2 h, 300 L/min)	24.5–25.5	93–96	No	3	3
12	28-Nov-17	11:00–13:00 (2 h, 300 L/min)	28.5–29.5	75–77	No	2	2
13	29-Aug-16	13:00–15:00 (2 h, 300 L/min)	31.0–32.0	70–80	No	1	1
14	30-Aug-16	13:00–15:00 (2 h, 300 L/min)	31.0–32.0	70–80	No	1	1
15	21-Sep-15	13:30–15:30 (2 h, 300 L/min)	31.0–32.0	63–70	Yes	1	1
Germany							
16	30-Jul-17	12:00–14:00 (2 h, 300 L/min)	12.0–15.0	80–83	No	2	2
Israel							
17	4-Jul-17	08:30–10:30 (2 h, 300 L/min)	35.0–39.0	36–38	No	2	2
Russia							
18	2-Dec-17	09:00–11:00 (2 h, 300 L/min)	−10.0–−15.0	78–80	No	1	1
19	3-Dec-17	15:00–17:00 (2 h, 300 L/min)	−10.0–−15.0	78–80	No	1	1
						**Total no. of samples (including blanks):**	**183**

### Temperature and relative humidity

Temperature (T) and relative humidity (RH) at the sampling site were measured using HOBO Temp/RH 2.5% Data Logger (Onset, USA).

### Filter processing, DNA extraction, quantitation and sequencing

All filter samples were subsequently processed for DNA extraction, quantitation, qPCR, metagenomic sequencing and computational analysis as described in our previous study^22^. In brief, the filter samples were first washed 3 times using 2 mL of phosphate-buffered saline (pH 7.2) with 0.1% (v/v) Triton X-100 assisted with water-bath sonication at room temperature for 1 min. After washing, the suspension liquid was concentrated onto a 0.02 µm Anodisc filter (Whatman, UK) using a vacuum manifold (DHI, Denmark). DNA was then extracted from the Anodisc with the DNeasy PowerWater kit (Qiagen, USA) following the manufacturer’s standard protocol with modifications to increase DNA yield^26^.

Final DNA solution was subjected for fluorometer quantification, qPCR and shotgun metagenomic sequencing. Fluorometer quantitation was measured with Qubit 2.0 (Invitrogen, USA) using the High Sensitivity double stranded DNA (HS dsDNA) kit. Taqman qPCR assays with universal bacterial (16S rRNA gene)^41^ and fungal (18S rRNA gene)^42^ primer set and probes were used to quantify the copy numbers of bacteria and fungi, respectively. The complete list of primers can be found in Table 2.

**Table 2:** List of primers and probes applied in the study.

Name	Sequence	Notes
16S 341F	5′-CCTACGGG**D**GGC**W**GCA-3′	Bacteria qPCR
16S 805R	5′-GGACTAC**HV**GGGT**M**TCTAATC-3′	Bacteria qPCR
Taqman probe	6FAM-5′-CAGCAGCCGCGGTA-3′-BBQ	Bacteria qPCR probe
FungiQuant-F	5′-GGRAAACTCACCAGGTCCAG-3′	Fungi qPCR
FungiQuant-R	5′-GSWCTATCCCCAKCACGA-3′	Fungi qPCR
FungiQuant-PrbLNA	6FAM-5′-TGGTGCATGGCCGTT-3′-BBQ	Fungi qPCR probe
16S 341F Illumina	5′-TCG TCG GCA GCG TCA GAT GTG TAT AAG AGA CAG CCT ACG GGN BGC ASC AG -3′	Amplicon for bacteria
16S 805R Illumina	5′-GTC TCG TGG GCT CGG AGA TGT GTA TAA GAG ACA GGG ACT ACH VGG GTW TCT AAT -3′	Amplicon for bacteria
ITS1F Illumina	5′- TCG TCG GCA GCG TCA GAT GTG TAT AAG AGA CAG CTT GGT CAT TTA GAG GAA GTA A -3′	Amplicon for fungi
ITS2R Illumina	5′- GTC TCG TGG GCT CGG AGA TGT GTA TAA GAG ACA GGC TGC GTT CTT CAT CGA TGC -3′	Amplicon for fungi

For direct metagenomic sequencing, libraries were prepared using Swift Biosciences’ Accel-NGS 2S Plus DNA Library kit following the standard protocol. All libraries were subsequently dual-barcoded with Swift Biosciences’ 2S Dual Indexing kit. PCR amplification selectively enriches for library fragments that have adapters ligated on both ends. The number of cycles were adjusted based on the starting amount of DNA (8–15 cycles). Upon pooling at equal volumes, libraries were sequenced on Illumina HiSeq2500 Rapid runs at a final concentration of 10–11 pM and a read-length of 251 bp paired-end (Illumina V2 Rapid sequencing reagents). Each ultra-low biomass sample was sequenced to a depth of at least two million paired-reads.

Raw reads from the sequencer were first trimmed from adapter sequences, low quality bases (<20 score) and short reads (<30 bp) using Cutadapt (v.1.8.1)^43^. The processed reads were then aligned against the NCBI’s NR database (v.25-02-2016) using RAPSearch2 (v.2.15)^44^. Results from the RAPSearch2 alignment were finally converted to read-match archive (rma) to be visualised with MEGAN5 software^33^.

### Experimental parameters optimisation

Important parameters for sampling, extraction and sequencing were tested and optimised based on absolute (fluorometer and qPCR) and relative abundance assessment (DNA sequencing). Importantly, it should be noted, that only samples collected at an identical time and location may be compared. Therefore, it is mandatory as an experimental setup to deploy multiple air samplers for each set of the parameter optimisation experiments. This is due to the high volatility of biomass concentration and composition of air, particularly when sampling at different time points throughout day and night. The replicability and robustness of this study was, therefore, enabled through simultaneous deployment of up to 12 air samplers at any given time (*n* = 12).

Comparison to other types of environmental samples: The ultra-low concentration of airborne biomass was investigated relative to other types of environmental samples. To negate possible differences due to sampling location and/or processing method, soil (1 gram per sample extraction), water (1 mL per sample extraction) and air samples (300 L/min, 2 h sampling duration) were collected within the same proximity (in Singapore) and were subsequently processed with identical protocol. Only DNA yield (ng/unit mass or volume of the samples) was assessed for this experiment.

The amassment parameters are sampling duration and sampling flow rate. *Sampling duration experiment*: With a fixed air flow rate (300 L/min, *n* = 3), sampling duration was varied at 15, 30, 60, 120 and 180 min. Further, multiple shorter duration samples were also compared to longer duration samples with matching time segments, i.e. first and second 15 min samples were compared to the matching 30 min sample. *Air flow rate experiment*: With a fixed duration (2 h), three groups of air samplers (*n* = 4) were run at the same time with varying flow rate at 100, 200 and 300 L/min. The experiments were assessed based on the impact of sampling duration and airflow variations on DNA quantity and microbial composition.

#### Sample storage experiment

Three sets of air samples collected simultaneously (300 L/min, 2 h, *n* = 4) were subjected to the following storage regimes; direct processing (fresh), −20 °C storage for 5 days (freezer) and room temperature storage for 5 days (RT) and compared for both DNA quantity and microbial profiles.

Parameters optimised for filter processing and DNA extraction were the use of sonication, detergent and impact of pre-incubation. *Sonication experiment*: Two sets of air samples collected at the same time (300 L/min, 2 h, *n* = 3) were subjected to filter washing with the room temperature water-bath sonication step included and excluded. *Detergent experiment*: Four sets of air samples collected at the same time (300 L/min, 2 h, *n* = 3) were washed with buffer containing four different concentrations of non-ionic detergent Triton-X 100 (%v/v): No detergent (0%), 0.01, 0.1 and 0.5%. *Pre-incubation experiment*: Three sets of air samples collected at the same time (300 L/min, 2 h, *n* = 4) were subjected to three different durations of pre-incubation in 55 °C water bath prior to proceeding with the subsequent lysis step of the DNA extraction. The durations were 1 h, 2 h and overnight (14–16 h). These durations were selected to enable the completion of the entire extraction process (filter washing and DNA extraction) within a standard working day (~8 h).

All the above experiments were assessed based on DNA quantity and microbial profiles of the resulting analysis.

The DNA sequencing result was evaluated for the DNA input amount, reproducibility, robustness and taxonomic classification difference between metagenomics and amplicon. *DNA input experiment*: From a given extracted DNA sample, four different DNA input amounts for direct metagenomic sequencing were tested: 10 ng, 5 ng, 2 ng and 0.5 ng. The number of PCR cycles during library construction were adjusted based on the DNA amount. The final result was assessed based on the taxonomic composition of the sequencing analysis. *Reproducibility between replicates*: A set of time series samples was analysed to investigate the similarity of the metagenomic profiles between the replicates. The time-series data contains twelve sets of time points with three replicates each. Each set was collected with 300 L/min flow rate and 2-hour sampling duration, spanning across 24 h.*Robustness across a range of climatic settings*: Air samples collected from locations with different climates (highly variables T and RH) were analysed regarding the success rate of DNA sequencing library construction due to varying amounts and quality of DNA input. 300 L/min flow rate and 2 h sampling duration were used to collect samples in Germany (temperate), Israel (dessert) and Russia (sub-arctic). *Comparison of shotgun metagenomic and amplicon marker gene sequencing*: The two sequencing approaches were evaluated using taxonomic assignments from identical sets of extracted air samples. DNA samples were split for shotgun metagenomic, 16S bacterial amplicon and ITS fungal amplicon sequencing. The sequencing and analysis methods for the bacterial and fungal amplicon sequencing are detailed in the following section.

### PCR-based amplicon sequencing and analysis

A subset of our ultra-low biomass samples were also subjected to amplicon sequencing for direct comparison with the shotgun metagenomic sequencing approach. For these samples, the first stage PCR was performed with the extracted genomic DNA as a template and the ITS1F-ITS2R^45^ primers for fungi and 16S 341F-805R^46^ primers for bacteria. Details of these primer sequences can be found in Table 2. KAPA HiFi HotStart master mix was used with a total reaction volume of 25 µL. For DNA input amount, 3 µL and 10 µL of DNA templates were used for fungi and bacteria, respectively. The cycling condition was 95 °C for 3 min, amplification cycles with 95 °C for 30 s, 65 °C for 30 s, 72 °C for 30 s, and a final extension at 72 °C for 5 min. The fungal samples were amplified with 15 cycles and the bacteria samples were amplified with 25 cycles. The PCR products were then purified with AMPure XP beads (Beckman Coulter) before performing the second stage PCR.

The second stage PCR (Indexing PCR) was performed according to the recommendations in Illumina’s “16S Metagenomic Sequencing Library Preparation” application note. This step uses a limited cycle PCR to complete the Illumina sequencing adapters and add dual-index barcodes to the amplicon target. Five microliters of the intermediate PCR product from the first stage PCR (Amplicon PCR) were used as template for the indexing PCR and samples were amplified with eight PCR cycles. Nextera XT v2 indices were used for dual-index barcoding to allow pooling of the amplicon targets for sequencing.

Finished amplicon libraries were quantitated using Promega’s QuantiFluor dsDNA assay and the average library size was determined on an Agilent Tapestation 4200. Library concentrations were then normalised to 4 nM and validated by qPCR on a QuantStudio-3 real-time PCR system (Applied Biosystems), using the Kapa library quantification kit for Illumina platforms (Kapa Biosystems). The libraries were then pooled at equimolar concentrations and sequenced on the Illumina MiSeq platform with 20% PhiX spike-in and at a read-length of 300 bp paired-end (MiSeq V3 reagents).

After sequencing, raw reads were first trimmed from adapter sequences, low-quality bases and short reads using Cutadapt (v.1.8.1)^43^. After trimming, the R1 and R2 reads were first paired with minimum overlap of 10 bp and subsequently aligned against UNITE ITS database (v.7.1) for the ITS sequences and SILVA 16S database (release 132) for the 16S sequences using command line blastn^36^ (version 2.2.28 + ). Results from blastn alignments were also converted to read-match archive (rma) format for visualisation with the MEGAN5 software to facilitate direct comparison with the metagenomic sequencing analysis. The default LCA parameters were used.

### Statistical analysis

For quantitative analysis from Qubit 2.0 Fluorometer and qPCR, all statistical tests were conducted with Mann–Whitney test. As mentioned previously, we acknowledge the limitations of these tests due to the relatively low number of observations (*n* = 3 or *n* = 4) for each set of samples. Due to the volatile nature of air sample, only samples collected at the same time and location can be directly compared. Thus, the number of replications was limited by the number of samplers which could be deployed at a given time (*n* = 12).

For metagenomic analysis, significant differences between groups of samples were mainly determined by ANOSIM test based on distance matrices between the samples compared. Distance matrices were created through PRIMER7 software based on taxa (genus level, cut-off at 0.05% of total assigned reads) read counts of each sample generated by MEGAN5. The distance matrix calculated based on Bray–Curtis algorithm was used to evaluate proportional difference (community structure) of the microbial communities between samples, while the distance matrix calculated based on Jaccard algorithm was used to determine presence–absence difference (community membership/richness) of different taxa detected in the compared group of samples. For reproducibility assessment among replicates, environmental time series data were used in which air samples with two-hourly time resolution were collected in 24 h with three replicates each. Similarity percentage (SIMPER) analysis was conducted with PRIMER7 software with the samples grouped based on the replicates.

### Blank sample collection and analysis

Five filter blank samples were collected and analysed. Filter blank samples were collected by attaching a clean, unused filter onto the air sampler at the sampling location and collecting them after 1 min without running the sampler. They were subjected to the same extraction methods and metagenomic analysis pipeline as the actual air samples.

### Reporting summary

Further information on research design is available in the Nature Research Reporting Summary linked to this article.

## Supplementary information

Supplementary Information

Reporting Summary

## Data Availability

All raw unprocessed reads have been submitted to NCBI under the bio-project accession number PRJNA638794.

## References

[1] Darwin, C. *The Voyage of the Beagle* (Cosimo Inc., 2008).

[2] Von Humboldt, A. & Aimé B. *Personal Narrative of Travels to the Equinoctial Regions of America: During the Years 1799-1804* (Cosimo Inc., 2013).

[3] Gilbert JA, Jansson JK, Knight R (2014). The Earth Microbiome project: successes and aspirations. BMC Biol..

[4] Silvia, C. M. & Stal. J. L. *The Marine Microbiome* (Springer International, 2016).

[5] Burrows SM, Elbert W, Lawrence MG (2009). Bacteria in the global atmosphere. Atmos. Chem. Phys..

[6] Bauer H (2002). The contribution of bacteria and fungal spores to the organic carbon content of cloud water, precipitation and aerosols. Atmos. Res..

[7] Prussin AJ, Garcia EB, Marr LC (2015). Total concentrations of virus and bacteria in indoor and outdoor air. Environ. Sci. Technol. Lett..

[8] Jones AM, Harrison RM (2004). The effects of meteorological factors on atmospheric bioaerosol concentrations—a review. Sci. Total Environ..

[9] Schulz-Bohm K, Martín-Sánchez L, Garbeva P (2017). Microbial volatiles: small molecules with an important role in intra- and inter-kingdom interactions. Front Microbiol.

[10] Misztal PK (2018). Emission factors of microbial volatile organic compounds from environmental bacteria and fungi. Environ. Sci. Technol..

[11] Bourdillon BYRB, Lidwell M, Thomas JC (1941). A slit sampler for collecting and counting air-borne bacteria. Epidemiol. Infect..

[12] Palmgren U, Ström G, Blomquist G, Malmberg P (1986). Collection of airborne micro-organisms on Nuclepore filters, estimation and analysis-CAMNEA method. J. Appl. Bacteriol..

[13] Yamamoto N (2012). Particle-size distributions and seasonal diversity of allergenic and pathogenic fungi in outdoor air. ISME J..

[14] Lang-Yona N (2012). Annual distribution of allergenic fungal spores in atmospheric particulate matter in the eastern mediterranean; A comparative study between ergosterol and quantitative PCR analysis. Atmos. Chem. Phys..

[15] Hospodsky D (2012). Human occupancy as a source of indoor airborne bacteria. PLoS ONE.

[16] Fu X (2020). Indoor microbiome, environmental characteristics and asthma among junior high school students in Johor Bahru, Malaysia. Environ. Int.

[17] Luhung I (2018). Exploring temporal patterns of bacterial and fungal DNA accumulation on a ventilation system filter for a Singapore university library. PLoS ONE.

[18] Amend AS, Seifert KA, Samson R, Bruns TD (2010). Indoor fungal composition is geographically patterned and more diverse in temperate zones than in the tropics. Proc. Natl Acad. Sci..

[19] Tringe SG (2008). The airbone metagenome in an indoor urban environment. PLoS ONE.

[20] Yooseph S (2013). A metagenomic framework for the study of airborne microbial communities. PLoS ONE.

[21] Cao C (2014). Inhalable microorganisms in Beijing’s PM_2.5_ and PM_10_ pollutants during a severe smog event. Environ. Sci. Technol..

[22] Gusareva ES (2019). Microbial communities in the tropical air ecosystem follow a precise diel cycle. Proc. Natl Acad. Sci..

[23] Ottesen EA (2014). Multispecies diel transcriptional oscillations in open ocean heterotrophic bacterial assemblages. Science.

[24] Kai W (2016). Ambient bioaerosol particle dynamics observed during haze and sunny days in Beijing. Sci. Total Environ..

[25] Dybwad M, Skogan G, Blatny JM (2014). Comparative testing and evaluation of nine different air samplers: end-to-end sampling efficiencies as specific performance measurements for bioaerosol applications. Aerosol Sci. Technol..

[26] Luhung I (2015). Protocol improvements for low concentration DNA-based bioaerosol sampling and analysis. PLoS ONE.

[27] Spring, A. M. et al. A method for collecting atmospheric microbial samples from set altitudes for use with next-generation sequencing techniques to characterize communities. *Air Soil Water Res.*10.1177/1178622118788871 (2018).

[28] Jiang W (2015). Optimized DNA extraction and metagenomic sequencing of airborne microbial communities. Nat. Protoc..

[29] Kim H, Park K, Lee M (2012). Biocompatible dispersion methods for carbon black. Toxicol. Res..

[30] Muthukumaran S (2005). The optimisation of ultrasonic cleaning procedures for dairy fouled ultrafiltration membranes. Ultrasonic Sonochem..

[31] Cragg MS (2003). Complement-mediated lysis by anti-CD20 mAb correlates with segregation into lipid rafts. Blood.

[32] Núñez A (2016). Monitoring of airborne biological particles in outdoor atmosphere Part 2: metagenomics applied to urban environments. Int. Microbiol..

[33] Huson DH, Auch AF, Qi J, Schuster SC (2007). MEGAN analysis of metagenomic data. Genome Res..

[34] Quast C (2012). The SILVA ribosomal RNA gene database project: improved data processing and web-based tools. Nucleic Acids Res..

[35] Abarenkov K (2010). The UNITE database for molecular identification of fungi–recent updates and future perspectives. N. Phytol..

[36] Altschul SF, Gish W, Miller W, Myers EW, Lipman DJ (1990). Basic local alignment search tool. J. Mol. Biol..

[37] Urich T (2008). Simultaneous assessment of soil microbial community structure and function through analysis of the meta-transcriptome. PLoS ONE.

[38] Dommergue A (2019). Methods to investigate the global atmospheric microbiome. Front. Microbiol..

[39] Spens J (2017). Comparison of capture and storage methods for aqueous macrobial eDNA using an optimized extraction protocol: advantage of enclosed filter. Methods Ecol. Evolution.

[40] Patterson EI (2020). Methods of inactivation of SARS-CoV-2 for downstream biological assays. J. Infect. Dis..

[41] Liu CM (2012). BactQuant: an enhanced broad-coverage bacterial quantitative real-time PCR assay. BMC Microbiol.

[42] Liu CM (2012). FungiQuant: a broad-coverage fungal quantitative real-time PCR assay. BMC Microbiol.

[43] Martin M (2011). Cutadapt removes adapter sequences from high-throughput sequencing reads. EMBnet J..

[44] Zhao Y, Tang H, Ye Y (2012). RAPSearch2: a fast and memory-efficient protein similarity search tool for next-generation sequencing data. Bioinformatics.

[45] Bokulich NA, Mills DA (2013). Improved selection of Internal Transcribed Spacer-specific primers enables quantitative, ultra-high-throughput profiling of fungal communities. Appl Environ. Microbiol..

[46] Takahashi S, Tomita J, Nishioka K, Hisada T, Nishijima M (2014). Development of a prokaryotic universal primer for simultaneous analysis of bacteria and archaea using next-generation sequencing. PLoS ONE.

