# Supplementary material for: Experimental parameters defining ultra-low biomass bioaerosol analysis
Source: NPJ Biofilms Microbiomes. 2021 Apr 16;7:37. doi: 10.1038/s41522-021-00209-4 (PMC8052325; doi:10.1038/s41522-021-00209-4)
Supplement: Supplementary file 1 — Supplementary Information [file 41522_2021_209_MOESM1_ESM.pdf]

Supplementary Materials for:

Experimental Parameters Defining Ultra-low Biomass Bioaerosol Analysis

Irvan Luhung[1#], Akira Uchida[1#], Serene Boon Yuean Lim[1], Nicolas E Gaultier[1], Carmon Kee[1], Kenny Lau[1], Elena Gusareva[1], Cassie E Heinle[1], Anthony Wong[1], BNV Premkrishnan[1], Rikky W Purbojati[1], Enzo Acerbi[1], Hie Lim Kim[1], Ana CM Junqueira[1,2], Sharon Longford[1], Sachin R. Lohar[1], Yap Zhei Hwee[1], Deepa Panicker[1], Koh Yanqing[1], Kavita K. Kushwaha[1], Ang Poh Nee[1], Alexander Putra[1], Daniela I. Drautz-Moses[1], Stephan C Schuster[1*]

[1] Singapore Centre for Environmental Life Sciences Engineering (SCELSE), Nanyang Technological University, Singapore

[2] Current address: Departamento de Genética, Instituto de Biologia, Universidade Federal do Rio de Janeiro, Rio de Janeiro, 21941-590 Brazil

*Correspondence to: scschuster@ntu.edu.sg

[#]These authors contributed equally to this work

Supplementary Discussion 1. qPCR for sampling duration experiment

Quantitative analysis with qPCR showed consistently increasing copy numbers of marker genes by function of sampling duration (Supplementary Fig.1). Within the tested range of 15 min to 3 hours of sampling duration, no adverse effect of longer air sampling on the filter medium was observed. Combination of the two 15-min sampling events yielded very similar quantity of marker genes to the 30-min sampling which started at 5:00 am. The same is true for the rest of the compared durations. Differences in copy numbers between the compared durations averaged at 21% with a range of 10-37% and 12-32% for bacterial and fungal DNA, respectively.

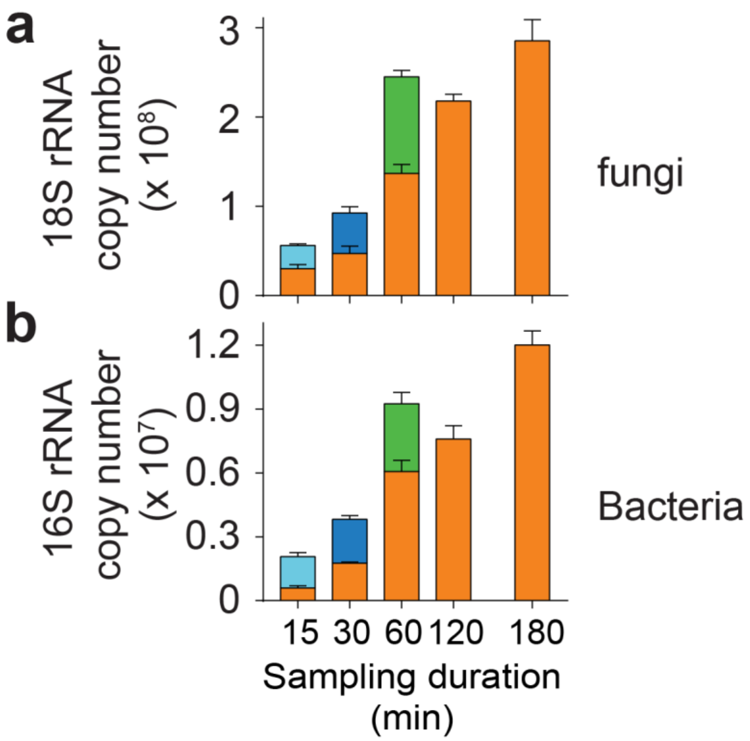

Supplementary Figure 1. qPCR analysis for sampling duration experiment. (a) Fungal 18S rDNA copy number and (b) bacterial 16S rDNA copy number of air samples taken with the same flowrate (300 L/min) but with different sampling duration. Samples taken in series are marked with different colours and stacked to allow for comparison with the corresponding sample that was collected with a single filter with the combined sampling duration. The bars represent mean values and the error bars were standard deviation among the replicates.

Supplementary Discussion 2. Impact of air sampling flowrate on biomass amassment and metagenomic profile

While more biomass could be amassed within a fixed sampling duration when a higher air flowrate was used, normalization of total DNA concentration and qPCR-based copy numbers with the total volume of sampled air (m^3) indicated up to 20% reduction in air sampling efficiency as the sampling flowrate was increased from 100 to 300 L/min (Supplementary Fig.2a). Possible causes include particle retention efficiency at different flowrates and/or nucleic acid damage due to stress from stronger air flow. For the purpose of maximizing time resolution of the intended analysis, higher sampling flowrate is preferred as the increase in biomass collection rate still outperforms the reduction in sampling efficiency. However, when accurate estimation of biomass concentration in a given space is the priority, air sampling with lower flowrate but longer duration may be considered for better biomass collection efficiency per unit volume of air.

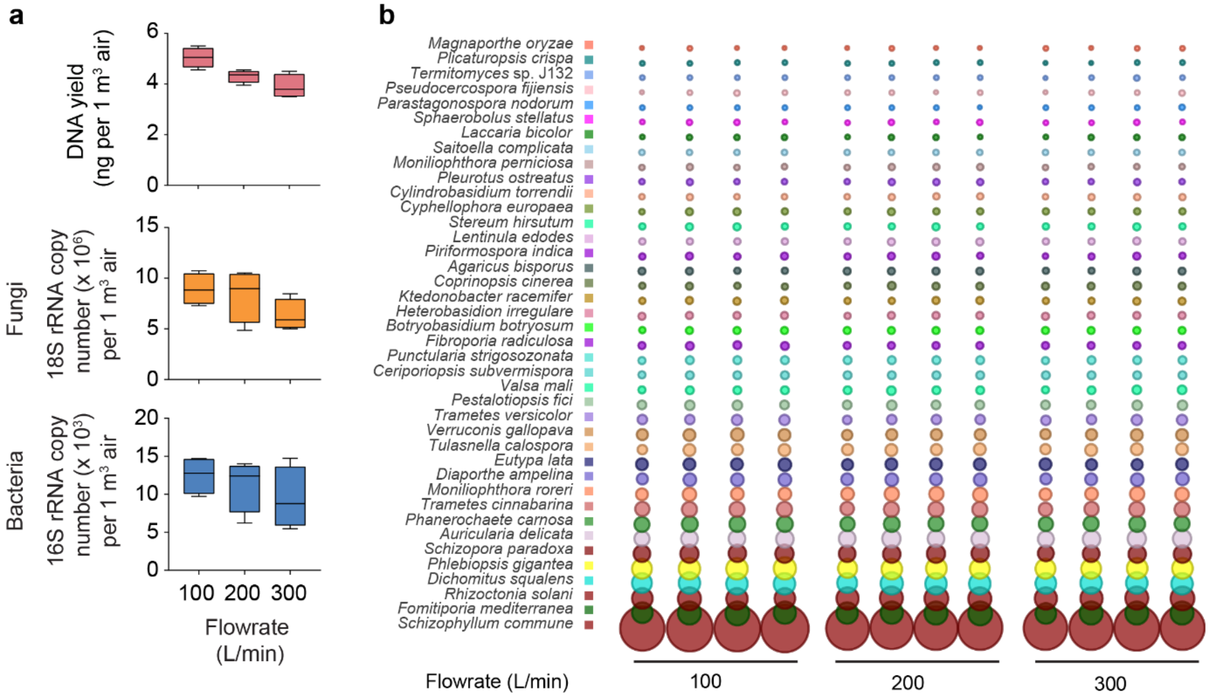

Supplementary Figure 2. Impact of air sampling flowrate. (a) Impact of air flowrate on biomass amassment efficiency normalized per m^3 of air, tested with DNA fluorometry and qPCR. The centre line, bound of box and whiskers represent median, 25th-75th percentile and min-to-max values respectively. (b) Taxonomic profile of the top 40 most abundant species for air samples collected with different flowrates.

The metagenomic analysis indicated no significant impact of the varying air sampling flowrates on the microbial composition of the air samples (Supplementary Fig.2b).

Supplementary Discussion 3. Impact of sample storage regimes on metagenomic analysis

Comparison between air samples stored in the freezer (-20°C) prior to processing and freshly processed samples did not reveal significant differences in the metagenomic profiles. Samples stored at room temperature (23°C), however, exhibited compositional differences compared to the fresh samples. After 5 days of room temperature storage, abundances of certain fungi, e.g. *Schizophyllum commune*, were slightly reduced (Supplementary Fig.3a). This change in composition, however, is relatively minor as the overall species ranking (based on relative abundances) is still maintained.

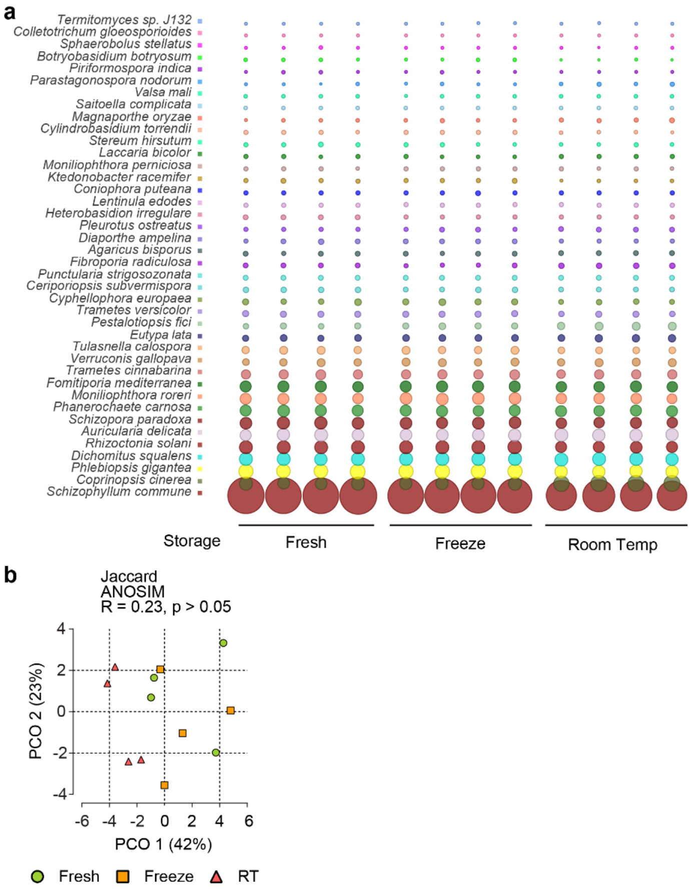

Supplementary Figure 3. Storage options of air filter samples. (a) Impact of storage condition on the taxonomic profile of the top 40 most abundant airborne species. (b) PCoA analysis based on Jaccard distance algorithm on species-level assignment for air samples subjected to different storage approaches.

Further beta diversity analysis on species richness (Jaccard) indicated no significant difference between the three storage regimes (Supplementary Fig.3b). This finding suggests that while proportional differences of certain species may be encountered, loss of species richness due to sample storage is unlikely.

Supplementary Discussion 4. Water-bath sonication at room temperature

Comparison of samples processed with and without 1-minute water-bath sonication at room temperature (RT) indicated no significant differences with regard to absolute and compositional abundance (Supplementary Fig.4). One possible consideration for maintaining this step is to reduce potential variation due to handling among different personnel processing the sample.

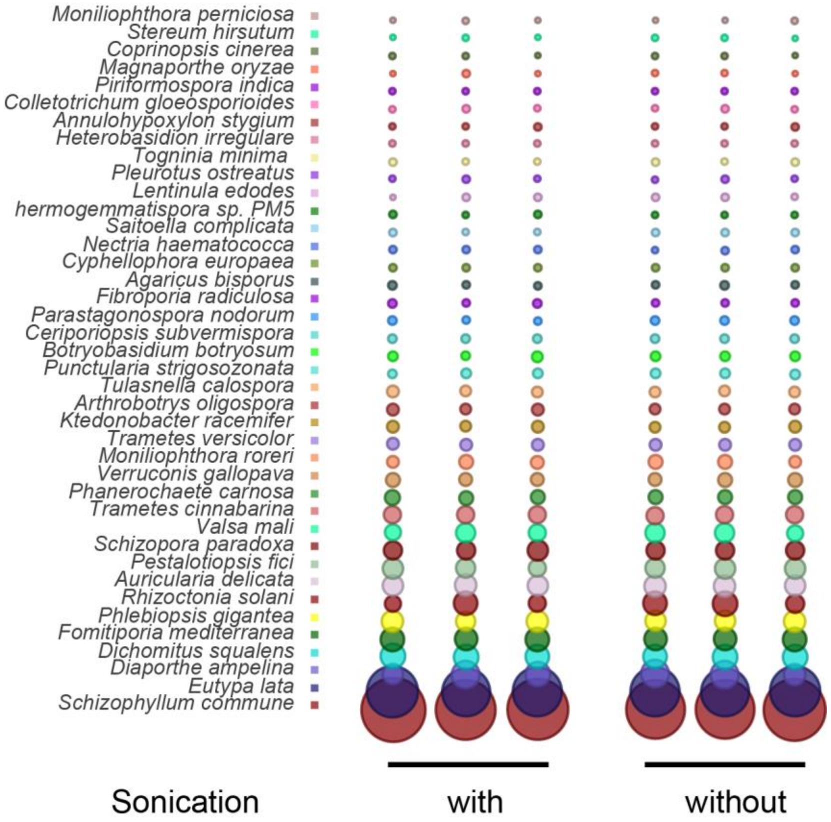

Supplementary Figure 4. Effect of water bath sonication at room temperature (RT). Taxonomic profile of the top 40 most abundant species from air samples extracted with and without the sonication step.

Supplementary Discussion 5. Filter wash buffer with Triton-X 100

Adding non-ionic detergent Triton-X 100 to the filter wash buffer substantially enhanced the detection of microbial taxa. The improvement is the most apparent for bacterial taxa (Supplementary Fig.5b), while having a less pronounced impact on fungal taxa (Supplementary Fig.5a). Beta diversity analysis on species richness (Supplementary Fig.5c) indicated significant differences in richness, mostly due to bacteria, only between the samples processed with and without the addtion of the detergent. Detergent concentration (%v/v) did not show a significant impact on species richness.

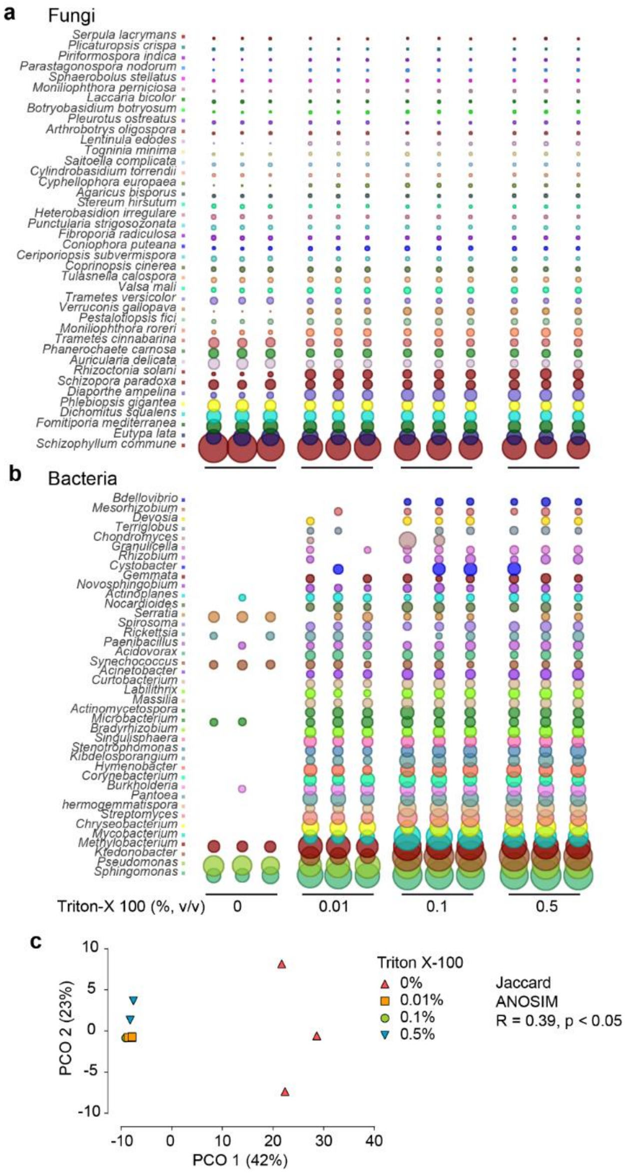

Supplementary Figure 5. Addition of Triton-X 100 to the extraction wash buffer. The taxonomic profile of the top 40 most abundant (a) fungal and (b) bacterial species from air samples extracted with filter wash buffer containing different %(v/v) concentrations of Triton-X 100. (c) PCoA based on Jaccard algorithm on species level assignment for air samples extracted with different %(v/v) concentrations of Triton-X 100.

<u>Supplementary Discussion 6. 16S rDNA and ITS sequences from metagenomic sequencing</u>

Taxonomic profiles generated from aligning amplicon sequencing reads and 16S/ITS reads extracted from metagenomic sequencing to the same database (SILVA for 16S and UNITE for ITS) were highly similar. For fungi, with both ITS from amplicon (Amp ITS) and ITS from the metagenomic dataset (WGS ITS) used as reference, the agreement between the two profiles ranged from 80-100% on genus level and 63-98% on species level for the top 40 most abundant taxa (Supplementary Fig. 6a).

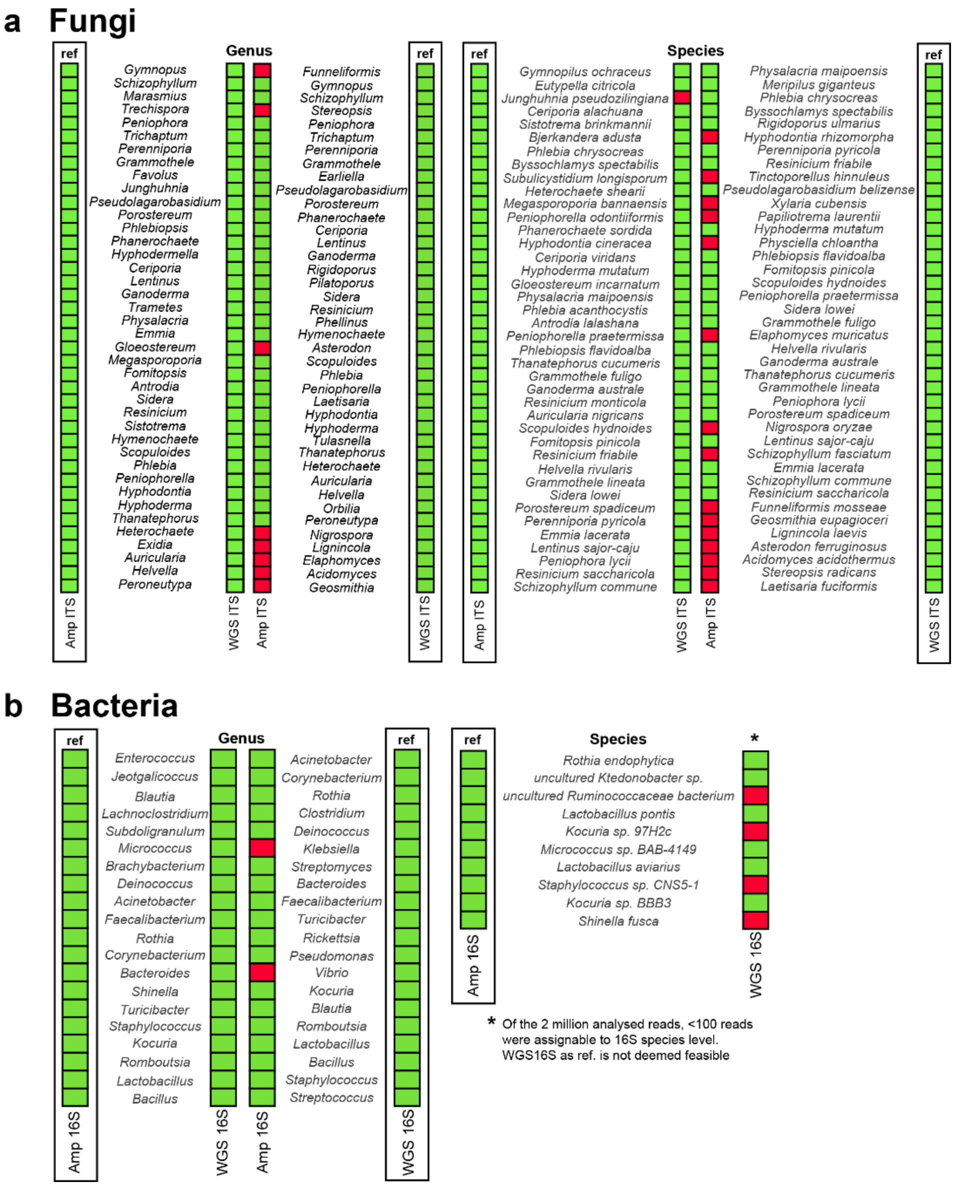

Supplementary Figure 6. Comparison of taxonomic assignment of the rDNA portion of metagenomic reads and 16S/ITS amplicon reads for the most abundant taxa. The metagenomic reads and the amplicon reads were produced from the same air DNA sample. The taxonomic assignments for (a) fungi and (b) bacteria are shown at genus as well as species level.

Of the two million metagenomic reads analysed per sample, only a few hundred could be identified as 16S sequences. This finding is consistent with the fact that ambient tropical air samples used for this study are substantially more abundant in fungi than bacteria. The limited number of 16S rDNA reads (WGS 16S) reduced the number of taxa that can be compared to the 16S amplicon sequencing result (Amp 16S). Despite this limitation, there were still 90-100% agreement between the two profiles for the top 20 most abundant bacterial genera and 60% agreement for the top 10 most abundant species (Supplementary Fig.6b). It must also be noted that differences between the two sequencing approaches are also partly caused by the fact that the amplicon sequencing targeted a certain part of the 16S/ITS gene based on the primers, while the rDNA reads from the metagenomic sequencing could come from any part of the 16S/ITS gene.

When the same database was used as reference, the high degree of agreement between the two sequencing approaches indicated that the discrepancies in taxonomic profiles between metagenomic and Amplicon (16S/ITS) sequencing pipelines were mainly attributed to biases in their respective public databases.

Supplementary Discussion 7. Sequencing of the blank samples

Prior to every sampling activity, all air samplers were cleaned and decontaminated by placing a sterile wipe wetted with cleaning solution (10% (v/v) bleach + 1% (v/v) NaOH + 0.1% (v/v) SDS + 80 mM $NaHCO_3$) on the metal grill where the air filter would be attached and allowing for a contact time of one minute. After removing the wipe, another sterile wipe wetted with 70% ethanol was placed on the metal grill, also for one minute. After removal of the ethanol wipe, the air filter was attached to the sampler.

To further ensure contamination control, filter blank samples were collected by placing an unused, clean filter on the air sampler for one minute at the sampling location after the initial cleaning procedure had been completed. Without running the sampler, the filter was then collected, transported and processed with the same pipeline as the other air samples.

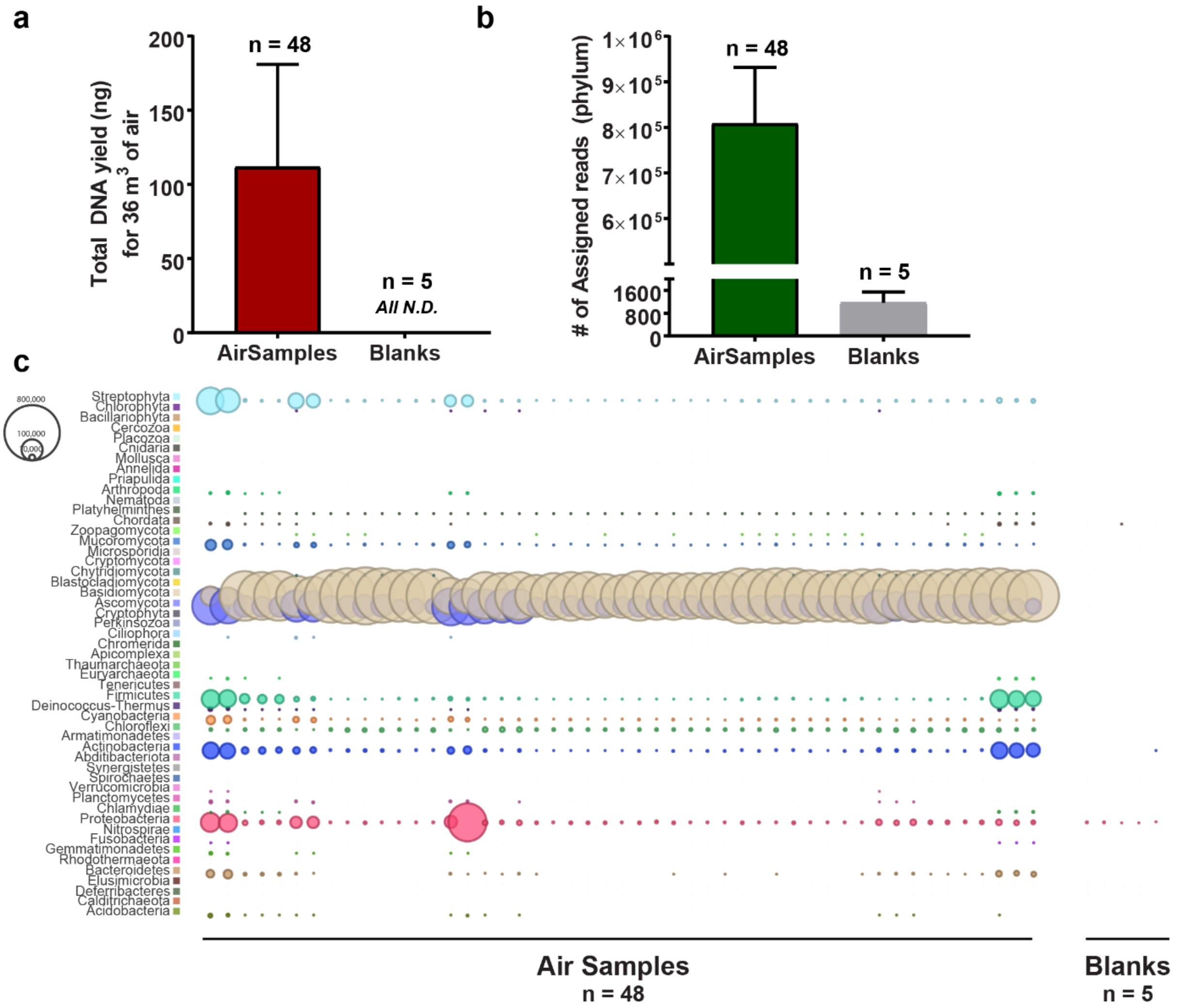

Supplementary Figure 7. Filter blank sample analysis. Comparison of air samples and filter blank samples in terms of (a) total DNA yield (fluorometry), (b) number of reads assignable at phylum level. The bars represent mean values and the error bars were standard deviation among the replicates. (c) the taxonomic profile of all phyla.

The results did not reveal any significant contamination during the sample collection and processing steps. First, DNA yield for all filter blank samples was below the detection limit of the fluorometer (Supplementary Fig.7a). Second, the sequencing results of the blank samples indicated that there were only very limited number of reads that could be meaningfully assigned at phylum level (< 2000 reads, Supplementary Fig.7b). Third, the most abundant taxa detected in the air samples were not observed in the blanks (Supplementary Fig.7c).

A number of human-associated taxa from the Proteobacteria phylum and homo genus were detected in the blank samples. Based on this reference, contaminating reads could be potentially identified and removed from our air samples by using Decontam (1). However, for samples used in this study, the number of identified contaminating reads were very low, and were removed by our implemented filter which required a minimum of 0.05% of assigned reads for taxa to be included in the downstream analysis.

Supplementary Reference

1. Davis NM, Proctor DM, Holmes SP, Relman DA and Callahan BJ. Simple statistical identification and removal of contaminant sequences in marker-gene and metagenomics data. Microbiome 2018; 6: 226.

Supplementary Discussion 8. Filter-based and liquid-based samplers

An air sampling campaign was carried out to compare high volumetric flowrate filter-based (SASS 3100) and liquid-based (Coriolis µ, Bertin, France) air samplers for the purpose of comparative metagenomic analysis. Sampling activities were conducted at identical time and location (Singapore, N1.346247, E103.679467) with 300 L/min flowrate. The experiment was repeated 3 times on 3 different days for replication purpose. All samples were then processed with the described ultra-low biomass pipeline for metagenomic sequencing.

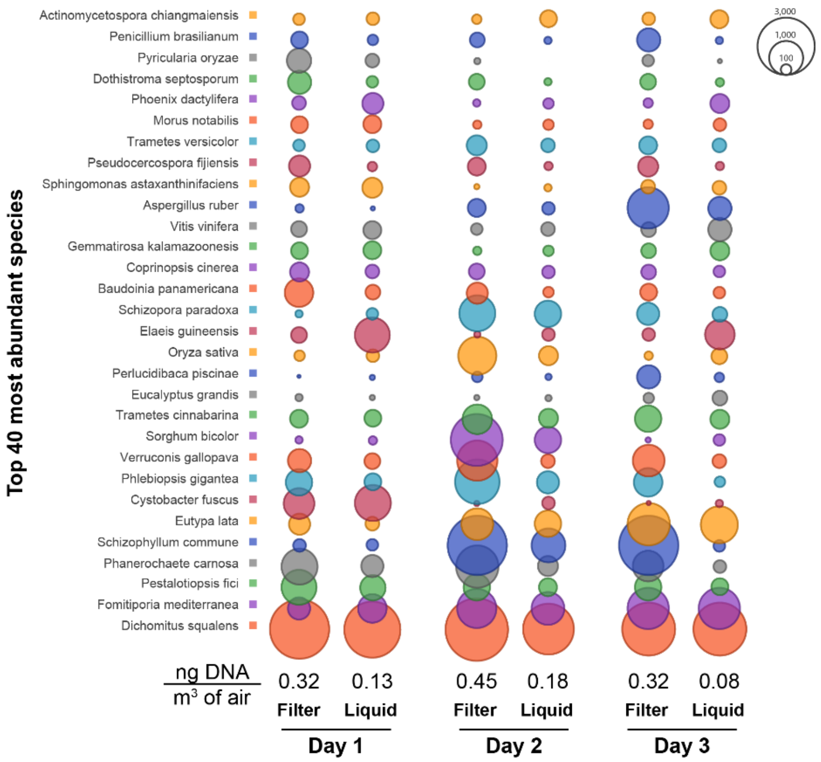

Supplementary Figure 8. Comparison of high volumetric flowrate filter- and liquid-based samplers. Bubble charts represent relative abundances (read counts) of the top 40 most abundant organisms. The DNA yield is presented as ng DNA / m^3 of air sampled.

The result (Supplementary Fig.8) indicated that both types of samplers produced highly similar metagenomic profiles of the air microbiome. All of the top 40 most abundant organisms (100%) were detected by both samplers on Day 1 and Day 3, while 98% (39/40) of them were captured by both sampling methods on Day 2. In terms of DNA yield, our filter-based sampler was found to consistently yielded higher DNA mass per volume of air sampled. This is likely because of the fact that the liquid media was constantly displaced or evaporated due to the high air flow introduced during sampling.

Supplementary Discussion 9. DNA accumulation rate comparison

Supplementary Table 1 Comparison of DNA accumulation rate for bioaerosol analysis pipelines

Study	Estimated DNA mass in 24 h of sampling (ng)	Sampling location (City) and rate (L/min)	Targeted particle size
Luhung et.al. (2021)	51.6 – 3,048	Singapore, 300 L/min	TSP
Jiang et al. (2015)	6.5 – 125	Beijing, 1,130 L/min	$PM_{2.5}$ and PM_{10}
Luhung et al. (2015)	2.2 – 4.0	Singapore, 16 L/min	TSP
Dommergue et al. (2019)	1.6 – 3.7	Grenoble, 500 - 1,167 L/min	TSP and PM_{10}

The above table summarizes published methods for bioaerosol sampling that report a measure of DNA yield. Despite its small size and portability, our filter-based sampler (SASS3100), in combination with the processing pipeline described in this study (*Luhung et.al. 2021),* was shown to result in the highest amassment rate of airborne biomass.

Supplementary Discussion 10. qPCR assays of DNA samples used in comparison between direct metagenomic and amplicon sequencing

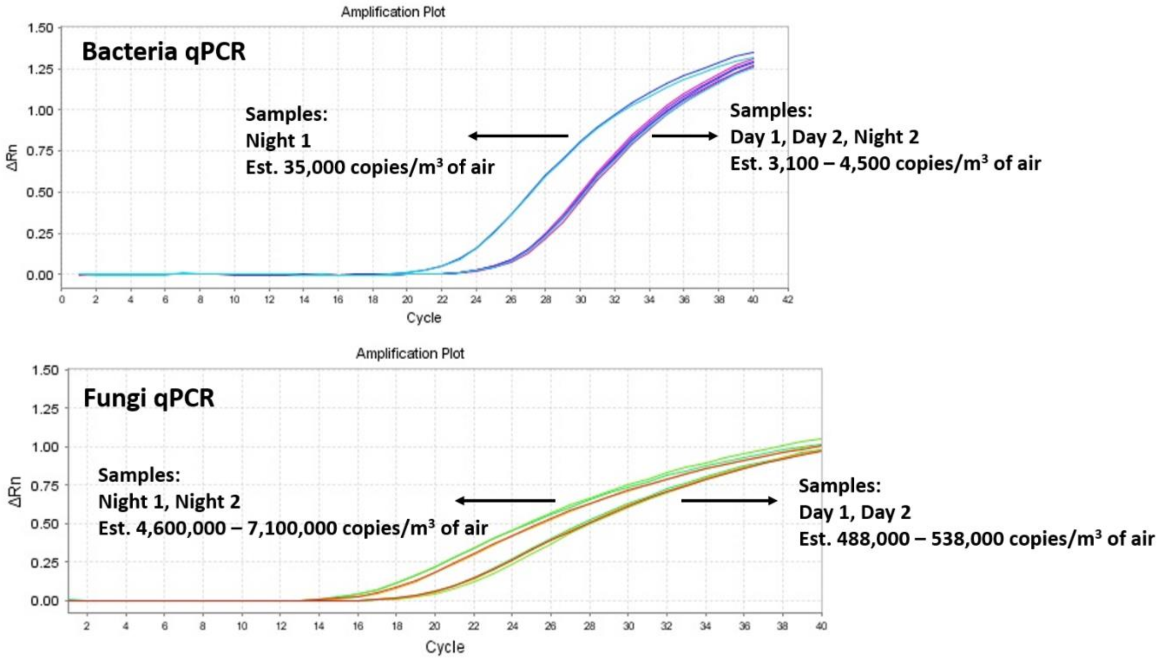

Supplementary Figure 9. qPCR assay for DNA samples specific to the dataset in Figure 6 (main manuscript).

A qPCR assay was conducted for four DNA samples (Day 1, Day 2, Night 1 and Night 2, with duplicates) specific to the dataset in Figure 6 (Supplementary Fig.9). The experiment determined the amounts of the 16S rDNA gene for Day 1, Day 2 and Night 2 air samples to be up to 10-fold lower than the one sample which was successfully sequenced (Night 1). In contrast, the copy numbers for fungi were abundant in all samples, resulting in successful sequencing. The analysis therefore indicates the threshold of detection for the 16S gene marker in amplicon-based sequencing assays.
