# Supplementary material for: Experimental parameters defining ultra-low biomass bioaerosol analysis
Source: NPJ Biofilms Microbiomes. 2021 Apr 16;7:37. doi: 10.1038/s41522-021-00209-4 (PMC8052325; doi:10.1038/s41522-021-00209-4)
Supplement: Supplementary file 2 — Reporting Summary [file 41522_2021_209_MOESM2_ESM.pdf]

nature research

Corresponding author(s): Stephan C. Schuster

Last updated by author(s): Feb 3, 2021

Reporting Summary

Nature Research wishes to improve the reproducibility of the work that we publish. This form provides structure for consistency and transparency in reporting. For further information on Nature Research policies, see our Editorial Policies and the Editorial Policy Checklist.

Statistics

For all statistical analyses, confirm that the following items are present in the figure legend, table legend, main text, or Methods section.

n/a	Confirmed	
☐	☒	The exact sample size (*n*) for each experimental group/condition, given as a discrete number and unit of measurement
☐	☒	A statement on whether measurements were taken from distinct samples or whether the same sample was measured repeatedly
☐	☒	The statistical test(s) used AND whether they are one- or two-sided<br>*Only common tests should be described solely by name; describe more complex techniques in the Methods section.*
☒	☐	A description of all covariates tested
☒	☐	A description of any assumptions or corrections, such as tests of normality and adjustment for multiple comparisons
☐	☒	A full description of the statistical parameters including central tendency (e.g. means) or other basic estimates (e.g. regression coefficient) AND variation (e.g. standard deviation) or associated estimates of uncertainty (e.g. confidence intervals)
☒	☐	For null hypothesis testing, the test statistic (e.g. *F*, *t*, *r*) with confidence intervals, effect sizes, degrees of freedom and *P* value noted<br>*Give P values as exact values whenever suitable.*
☒	☐	For Bayesian analysis, information on the choice of priors and Markov chain Monte Carlo settings
☒	☐	For hierarchical and complex designs, identification of the appropriate level for tests and full reporting of outcomes
☐	☒	Estimates of effect sizes (e.g. Cohen's *d*, Pearson's *r*), indicating how they were calculated

Our web collection on statistics for biologists contains articles on many of the points above.

Software and code

Policy information about availability of computer code

Data collection

1. FastQC (version 0.11.5) to check quality of reads from the sequencer
2. Cutadapt (version 1.8.1) to refine the sequencing data such as removing adapter sequences in the reads and bases with the quality score less than twenty (<20) and read lengths less than 30 bp
3. RAPSearch2 (version 2.15) to align sequence data against NR-database. Nucleotide sequence was converted to amino acid sequence to align against NR-database.
4.MEGAN software (version 5.11.3) to visualize metagenomic data using alignment data by RapSearch2
5.QIIME to pair trimmed R1 and R2 reads from Illumina sequencer
6.Command line BLASTn (version 2.2.23+) to align reads processed with QIIME against UNITE ITS and SILVA 16S databases.
7.PRIMER software v.7.0.13 (PRIMER-e, New Zealand) for Statistical analysis

Data analysis

Raw reads were checked using FastQC (version 0.11.5). Adapter sequences, bases with the quality score less than twenty (<20) and Reads with less than 30 bp in length were removed using Cutadapt (version 1.8.1).

For metagenomic sequencing, the trimmed reads were aligned against NCBI's NR (non-redundant) database. NR-database is a collection of all non-redundant GenBank CDS translations, Protein Data Bank, SwissProt, PIR, PRF excluding environmental samples from WGS projects. Alignment against NR-database was done using RAPSearch2 (version 2.15) with default parameters21. RAPSearch2 allows nucleotide sequences to be matched against protein sequences.

Results from the RAPSearch2 alignment were converted to read-match archive (rma) format to be visualized with MEGAN version 5 software. Parameters used while converting to 'rma' formats were, minimum support = 25, minimum score = 100, maximum matches = 25, and minimum complexity = 0.33. MEGAN uses lowest common ancestor (LCA) algorithm to predict the taxonomical relationship between species.

For amplicon sequencing, the trimmed R1 and R2 reads were first paired using Qiime with minimum overlap of 10 bp and subsequently aligned against UNITE ITS database for the ITS sequences and SILVA 16S database for the 16S sequences using command line blastn (version

2.2.23+). Results from blastn alignments were also converted to read-match archive (rma) format for visualization with MEGAN version 5. The default LCA parameters were used for the conversion

For manuscripts utilizing custom algorithms or software that are central to the research but not yet described in published literature, software must be made available to editors and reviewers. We strongly encourage code deposition in a community repository (e.g. GitHub). See the Nature Research guidelines for submitting code & software for further information.

Data

Policy information about availability of data

All manuscripts must include a data availability statement. This statement should provide the following information, where applicable:

- Accession codes, unique identifiers, or web links for publicly available datasets
- A list of figures that have associated raw data
- A description of any restrictions on data availability

All data is available within the submitted manuscript.

For both metagenomic and amplicon sequencing, all unprocessed raw reads have been uploaded to NCBI under the bio-project accession number PRJNA638794

Field-specific reporting

Please select the one below that is the best fit for your research. If you are not sure, read the appropriate sections before making your selection.

☐ Life sciences ☐ Behavioural & social sciences ☒ Ecological, evolutionary & environmental sciences

For a reference copy of the document with all sections, see nature.com/documents/nr-reporting-summary-flat.pdf

Ecological, evolutionary & environmental sciences study design

All studies must disclose on these points even when the disclosure is negative.

Study description	Development of pipeline to study the air microbiome with shotgun metagenomic and/or amplicon sequencing approaches at high temporal and taxonomic resolution.<br><br>Assessments for each of the investigated parameters were based on quantitative (fluorometer and qPCR) and qualitative analysis (DNA sequencing) using up to 12 units of air samplers.
Research sample	The method optimization was based on actual environmental samples. All of our samples were collection of total suspended particles (TSP) at the specified sampling location captured on a filter medium. We did not generate controlled air sample by means of, for instance, aerosolization of a known set of substances.
Sampling strategy	The majority of the sampling campaigns were conducted at an open balcony in a university campus in Singapore. These samples, therefore, represent ambient air from a site with tropical climate.<br><br>Sampling details: Multiple air samplers (SASS 3100) were placed approximately 1 meter apart from each other to avoid competition in air collection. Prior to sampling, the air samplers were first disinfected with lab cleaner solution (contain bleach) and 70% ethanol using dust-free laboratory tissue (Kim wipe). After pre-running the air samplers for 1 minute to dry residual ethanol, sterile filter (SASS bioaerosol filter) was attached onto the sampler and the sampler is turned on. After sampling, the samples were immediately processed, or otherwise stored in -20˚C freezer until sample processing.<br><br>Additional field samplings were conducted in Israel, Germany and Russia to test the robustness of the air sampling pipeline in different climates. In these occasions, air samplers (2 units) were set 50-100 cm apart (depends on the space availability). The samples from the field trips were not frozen to avoid freezing and thawing. They were kept under room temperature until the samples were brought back to the lab and processed.<br><br>Due to the volatile nature of air samples, only samples collected simultaneously could be compared in varying investigated experimental parameters. For this reason, the number of replicates for each investigated groups/conditions was limited by the number of air samplers that could be deployed simultaneously at the time. The experiments employed triplication or quadruplication for four or three investigated variables respectively.
Data collection	Sampling location (GPS coordinates), date, sampling time (start and duration) were recorded at the time of sampling. Temperature and relative humidity was obtained by HOBO logger.
Timing and spatial scale	For sampling with the purpose of comparing day and night air samples, sampling time was standardized to noon and midnight (+- 1 hour). Unless stated otherwise, most air samples were collected with 2 h sampling duration.<br><br>In addition to Singapore as our base sampling location, we also conducted sampling in Israel, Germany and Russia which have different climatic conditions to test the robustness of our sampling and analysis approach.
Data exclusions	There was no data excluded from the presentation.
Reproducibility	Similarity Percentage (SIMPER) analysis conducted on sequencing datasets used in the current method development study and environmental time series datasets (Gusareva et al. 2019) which utilized the same sampling and processing pipeline indicated an

average similarity percentage of 91% among the replicates. The sample replicates referred here are individual/separate filter samples collected at the same location and at the same time. For each sample group, there are always three to four replicates.

Gusareva, E. S. et al. Microbial communities in the tropical air ecosystem follow a precise diel cycle. Proc. Natl. Acad. Sci. 116, 23299–23308 (2019)

Randomization

Twelve air samplers were randomly placed within the vicinity of the sampling location for every sampling event. For each parameter optimization, samples collected from the sampling site were randomly chosen and grouped for the intended parameter testing.

Blinding

Blinding was not relevant to our study because all the samples were handled equally through the pipeline.

Did the study involve field work? ☒ Yes ☐ No

Field work, collection and transport

Field conditions

In addition to Singapore as our base sampling location in a tropical climate region, sampling was also conducted in Israel, Russia and Germany to test the robustness of our pipeline. Israel represents dry climate, Russia was for cold climate, while Germany was for temperate climate.

Location

Singapore: sampling location at N1.3462, E103.6794, on top floor of a 4-story building with roof balcony.

Germany: sampling location at N47.5798, E9.6789 (balcony of residential house)

Russia: Sampling location at N55.7110, E84.9370 (balcony of residential house)
Collaborator: Prof. Vadim A. Stepanov, Research Institute of Medical Genetics, Tomsk National Medical Research Centre, Russian Academy of Sciences, Tomsk, Russia

Israel: Sampling location at N31.1943 E35.3588
Collaborator: Prof. Shmuel C. Shapira MD MPH, Institute for Biological Research, Israel

Access & import/export

Samples were collected by our research staff. No permission was required for sampling and import/export at the customs.

Disturbance

No disturbance caused. The air samplers do not emit any harmful chemical. Noise from samplers were less than 50 dB per unit within 1 meter distance and sampling was performed without the presence of human occupant nearby.

Reporting for specific materials, systems and methods

We require information from authors about some types of materials, experimental systems and methods used in many studies. Here, indicate whether each material, system or method listed is relevant to your study. If you are not sure if a list item applies to your research, read the appropriate section before selecting a response.

Materials & experimental systems

n/a	Involved in the study	
☒	☐	Antibodies
☒	☐	Eukaryotic cell lines
☒	☐	Palaeontology and archaeology
☒	☐	Animals and other organisms
☒	☐	Human research participants
☒	☐	Clinical data
☒	☐	Dual use research of concern

Methods

n/a	Involved in the study	
☒	☐	ChIP-seq
☒	☐	Flow cytometry
☒	☐	MRI-based neuroimaging
